# Evaluation of Reliability and Correlations of Quality Measures in Cancer Care

**DOI:** 10.1001/jamanetworkopen.2021.2474

**Published:** 2021-03-22

**Authors:** Nancy L. Keating, Jessica L. F. Cleveland, Alexi A. Wright, Gabriel A. Brooks, Laurie Meneades, Lauren Riedel, Jose R. Zubizarreta, Mary Beth Landrum

**Affiliations:** 1 Department of Health Care Policy, Harvard Medical School, Boston, Massachusetts; 2 Division of General Internal Medicine, Brigham and Women’s Hospital, Boston, Massachusetts; 3 Department of Informatics and Analytics, Dana-Farber Cancer Institute, Boston, Massachusetts; 4 Department of Medical Oncology, Dana-Farber Cancer Institute, Boston, Massachusetts; 5 Division of Population Sciences, Dana-Farber Cancer Institute, Boston, Massachusetts; 6 Section of Medical Oncology, Dartmouth-Hitchcock Medical Center, Lebanon, New Hampshire; 7 Department of Biostatistics, Harvard T.H. Chan School of Public Health, Boston, Massachusetts; 8 Department of Statistics, Harvard Faculty of Arts and Sciences, Cambridge, Massachusetts

## Abstract

**Importance:**

Measurement of the quality of care is important for alternative payment models in oncology, yet the ability to distinguish high-quality from low-quality care across oncology practices remains uncertain.

**Objective:**

To assess the reliability of cancer care quality measures across oncology practices using registry and claims-based measures of process, utilization, end-of-life (EOL) care, and survival, and to assess the correlations of practice-level performance across measure and cancer types.

**Design, Setting, and Participants:**

This cross-sectional study used the Surveillance, Epidemiology, and End Results (SEER) Program registry linked to Medicare administrative data to identify individuals with lung cancer, breast cancer, or colorectal cancer (CRC) that was newly diagnosed between January 1, 2011, and December 31, 2015, and who were treated in oncology practices with 20 or more patients. Data were analyzed from January 2018 to December 2020.

**Main Outcomes and Measures:**

Receipt of guideline-recommended treatment and surveillance, hospitalizations or emergency department visits during 6-month chemotherapy episodes, care intensity in the last month of life, and 12-month survival were measured. Summary measures for each domain in each cohort were calculated. Practice-level rates for each measure were estimated from hierarchical linear models with practice-level random effects; practice-level reliability (reproducibility) for each measure based on the between-measure variance, within-measure variance, and distribution of patients treated in each practice; and correlations of measures across measure and cancer types.

**Results:**

In this study of SEER registry data linked to Medicare administrative data from 49 715 patients with lung cancer treated in 502 oncology practices, 21 692 with CRC treated in 347 practices, and 52 901 with breast cancer treated in 492 practices, few practices had 20 or more patients who were eligible for most process measures during the 5-year study period. Patients were 65 years or older; approximately 50% of the patients with lung cancer and CRC and all of the patients with breast cancer were women. Most measures had limited variability across practices. Among process measures, 0 of 6 for lung cancer, 0 of 6 for CRC, and 3 of 11 for breast cancer had a practice-level reliability of 0.75 or higher for the median-sized practice. No utilization, EOL care, or survival measure had reliability across practices of 0.75 or higher. Correlations across measure types were low (*r* ≤ 0.20 for all) except for a correlation between the CRC process and 1-year survival summary measures (*r* = 0.35; *P* < .001). Summary process measures had limited or no correlation across lung cancer, breast cancer, and CRC (*r* ≤ 0.16 for all).

**Conclusions and Relevance:**

This study found that quality measures were limited by the small numbers of Medicare patients with newly diagnosed cancer treated in oncology practices, even after pooling 5 years of data. Measures had low reliability and had limited to no correlation across measure and cancer types, suggesting the need for research to identify reliable quality measures for practice-level quality assessments.

## Key Points

**Question:**

Can the quality of care be assessed reliably across medical oncology practices, and what are the correlations of different types of practice-level performance across quality measure and cancer types?

**Findings:**

In this cross-sectional study, registry and claims-based measures of care processes, utilization, end-of-life care, and survival for newly diagnosed fee-for-service Medicare beneficiaries with cancer were limited by small numbers of patients across oncology practices, even after pooling 5 years of data. Measures of care quality had low reliability and had limited to no correlation across measure and cancer types.

**Meaning:**

Results of this study suggest that additional research is needed to identify reliable quality measures for practice-level assessments of quality.

## Teaser

This cross-sectional study assesses measures of care process, utilization, end-of-life care, and survival at oncology practices that treat older Medicare beneficiaries with newly diagnosed lung, breast, or colorectal cancer.

## Introduction

Value-based payment models aim to improve care quality, enhance patient experience, and lower spending on care. Payers are developing more models for specialty care; for example, the Centers for Medicare & Medicaid Services (CMS) Oncology Care Model (OCM) focuses on care for patients with cancer who are undergoing chemotherapy.^1^ The OCM and other value-based payment models typically measure spending and care quality at the practice level. However, in addition to challenges in identifying high-quality oncology care,^2,3^ relatively little is understood about identifying high-quality care at the practice level. The OCM currently incorporates 6 measures of quality for calculating performance-based payments, including claims-based measures of emergency department (ED) visits and hospice use.^4^ For oncology practices that do not participate in the OCM or another advanced alternative payment model, the Medicare Access and CHIP Reauthorization Act requires quality reporting through the Merit-based Incentive Payment System.^5^ National organizations, such as the American Society of Clinical Oncology (ASCO), the American College of Surgeons Commission on Cancer, the National Quality Forum, and CMS, have developed and/or compiled measure sets to assess oncology care quality,^6,7,8,9^ although the performance of these measure sets has not typically been assessed at the practice level.

In this cross-sectional study, we characterized practice-level quality of oncology care using registry and claims-based measures of process, utilization, end-of-life (EOL) care, and survival for older patients with newly diagnosed lung cancer, colorectal cancer (CRC), or breast cancer. We then assessed the reliability of cancer care quality measures across oncology practices. We also assessed the correlations of practice-level performance across types of measures and cancers to ascertain whether practices with better performance in some types of measures or cancers had similarly better performance in other types.

## Methods

This cross-sectional study used the Surveillance, Epidemiology, and End Results (SEER) Program cancer registry linked to Medicare administrative data from January 1, 2010, to December 31, 2016 (the most recent dates in which data were available), to obtain data on cancers diagnosed between January 1, 2011, and December 31, 2015.^10^ The population-based SEER registry includes demographic and clinical information for all patients with newly diagnosed cancer who were living in areas that covered 28% of the US population during the study period.^11^ Medicare data included inpatient, outpatient, carrier (including fee-for-service claims submitted by those that provide services, such as physicians, physician assistants, clinical social workers, nurse practitioners, and organizations), durable medical equipment, and hospice and home health files. This study was approved by the Harvard Faculty of Medicine Institutional Review Board, which granted a waiver of informed consent because the study was a secondary analysis of previously collected data. We followed the Strengthening the Reporting of Observational Studies in Epidemiology (STROBE) reporting guideline.

### Study Population and Quality Measures

We included individuals with lung cancer, CRC, or breast cancer (all histologic types) that was diagnosed between January 1, 2011, and December 31, 2015. Individuals were 65 years or older, were continuously enrolled in Parts A and B of fee-for-service Medicare (non–health maintenance organization), and were alive through at least 6 months after diagnosis, although some measures required longer continuous enrollment (eTable 1 in the Supplement). Measures of adjuvant endocrine therapy for breast cancer required Part D enrollment. See eTable 1 in the Supplement for cohort identification.

Quality measures (Table 1) included care processes, ED visits, and hospital use for patients who were receiving chemotherapy as well as EOL care and survival (lung cancer and CRC only, given high breast cancer–specific survival) that could be assessed using registry and administrative claims data. Quality measures were adapted from those endorsed by the National Quality Forum,^8^ American College of Surgeons,^7^ ASCO Quality Oncology Practice Initiative,^6^ OCM,^4^ CMS Consensus Core Set,^9^ or National Comprehensive Cancer Network or ASCO guidelines that were applicable during the study period and for which we anticipated sufficient sample sizes. eAppendix 3 and eTable 5 in the Supplement detail the measure specifications.

**Table 1.** Reliability of Measures Across Practices

Measure	Source of measure	No. of practices with >20 patients	Median (interquartile range)		
			Rate across practices, %^a^	No. of patients in practice	Measure Reliability
**Process**					
Lung cancer processes (n = 502 total practices)					
Radiation for stage I or II NSCLC without surgery	NCCN	32	23 (12-30)	28 (24-35)	0.74 (0.71-0.78)
Adjuvant chemotherapy for stage II-IIIA NSCLC with surgery	NCCN	37	78 (75-83)	27 (22-31)	0.60 (0.55-0.64)
Chemotherapy ≤60 d of surgery for resected stage II-IIIA NSCLC	NCCN; QOPI 81	20	88 (86-90)	24 (20-34)	0.34 (0.30-0.42)
Chemotherapy and radiation for stage IIIA NSCLC	NCCN	12	95^b^	25 (21-34)	NA
Surveillance chest computed tomography for resected stage I, II, or IIIA NSCLC	NCCN	111	75 (67-80)	29 (23-42)	0.74 (0.69-0.80)
Lung cancer process summary	NA	191	58 (53-62)	34 (26-49)	0.57 (0.51-0.66)
CRC processes (n = 347 total practices)					
Adjuvant chemotherapy for stage III colon cancer	NQF 0223; CMS Consensus Core Set	84	71 (69-73)	30 (24-43)	0.36 (0.30-0.44)
Chemotherapy ≤60 d of surgery for resected stage III colon cancer	NCCN; QOPI 68	47	77 (74-79)	28 (24-40)	0.31 (0.28-0.39)
Surveillance colonoscopy for resected stage I-III CRC	NCCN	223	35 (34-36)	36 (25-55)	0.02 (0.02-0.04)
Surveillance carcinoembryonic antigen for stage II or III CRC	NCCN	184	91 (89-92)	36 (25-50)	0.42 (0.33-0.50)
Surveillance computed tomography for resected stage II or III CRC	NCCN	184	44 (37-53)	36 (25-50)	0.74 (0.66-0.80)
CRC process summary	NA	227	55 (54-57)	36 (25-56)	0.34 (0.26-0.44)
Breast cancer processes (n = 492 total practices)					
Radiation					
After breast-conserving surgery	NQF 219	328	75 (68-81)	49 (30-92)	0.78 (0.68-0.87)
After mastectomy for high-risk cancers, range	CoC MASTRT	2	75^b^	21-51	NA
Adjuvant chemotherapy for stage I (T1c) to III, ER/PR-negative cancers, range	NQF 0559; QOPI 53; CMS Consensus Core Set	2	84^b^	23-28	NA
Chemotherapy ≤60 d of surgery for stage I-III breast cancer		16	81 (77-86)	27 (23-38)	0.52 (0.48-0.61)
Adjuvant endocrine therapy for ER or PR-positive breast cancer	NQF 0223; QOPI 59	251	88 (86-89)	41 (28-65)	0.41 (0.32-0.52)
*ERBB2* (formerly *HER2*) assessed at diagnosis	QOPI 54	492	92 (91-94)	64 (36-124)	0.76 (0.64-0.86)
Trastuzumab received					
When *ERBB2* positive	QOPI 57; NQF 1858; CMS Consensus Core Set	66	55 (49-60)	29 (24-40)	0.57 (0.52-0.64)
When *ERBB2* negative or unknown^c^	NQF 1857; CMS Consensus Core Set	462	0.6 (0.5-0.6)	59 (34-112)	0.08 (0.05-0.15)
Intravenous bisphosphonates or denosumab if bone metastases	QOPI 61	5	56^b^	24 (20-27)	0.04 (0.03-0.04)^c^
Positron emission tomography scan, computed tomography, or bone scan at diagnosis for stage I or II breast cancer; low value or lower score better	QOPI 62a	437	21 (15-29)	57 (33-106)	0.87 (0.79-0.92)
Surveillance mammography for stage I or II breast cancer	NCCN	432	56 (52-59)	57 (33-106)	0.55 (0.42-0.70)
Breast cancer process summary	NA	492	64 (62-66)	64 (36-124)	0.67 (0.53-0.79)
**Utilization**					
Lung cancer utilization (n = 421 total practices)					
Hospitalizations during chemotherapy episodes	OCM 1	421	45 (43-48)	80 (47-144)	0.48 (0.35-0.63)
ED visits during 6-mo chemotherapy episodes that did not lead to hospital stay	OCM 2	421	16 (15-18)	80 (47-144)	0.37 (0.26-0.52)
Lung cancer utilization summary	NA	421	59 (57-62)	80 (47-144)	0.45 (0.32-0.59)
CRC utilization (n = 262 total practices)					
Hospitalizations during chemotherapy episodes	OCM 1	262	37 (36-39)	70 (49-113)	0.29 (0.22-0.40)
ED visits during 6-mo chemotherapy episodes that did not lead to hospital stay	OCM 2	262	16 (15-17)	70 (49-113)	0.35 (0.28-0.47)
CRC utilization summary	NA	262	50 (49-52)	70 (49-113)	0.36 (0.29-0.48)
Breast cancer utilization (n = 498 total practices)					
Hospitalizations during chemotherapy episodes	OCM 1	498	13 (12-14)	134 (73-287)	0.35 (0.22-0.53)
ED visits during 6-mo chemotherapy episodes that did not lead to hospital stay	OCM 2	498	12 (11-13)	134 (73-287)	0.40 (0.27-0.59)
Breast cancer utilization summary	NA	498	24 (23-25)	134 (73-287)	0.43 (0.29-0.61)
All cancers utilization (n = 701 total practices)					
Hospitalizations during chemotherapy episodes	OCM 1	701	25 (24-27)	142 (64-376)	0.51 (0.32-0.74)
ED visits during 6-mo chemotherapy episodes that did not lead to hospital stay	OCM 2	701	14 (13-15)	142 (64-376)	0.49 (0.30-0.72)
All cancers utilization summary	NA	701	38 (36-39)	142 (64-376)	0.47 (0.29-0.70)
**EOL care**					
Lung cancer (n = 397 total practices)					
Proportion of patients who died who did not enroll in hospice >3 d before death	NQF 0215; NQF 0216; CMS Consensus Core Set	397	47 (43-51)	49 (31-93)	0.55 (0.44-0.70)
>1 ED visit in last 30 d of life	NQF 0211; CMS Consensus Core Set	397	22 (21-24)	49 (31-93)	0.27 (0.19-0.41)
ICU admission in last 30 d of life	NQF 0213; CMS Consensus Core Set	397	31 (27-35)	49 (31-93)	0.57 (0.46-0.72)
Chemotherapy in last 2 wk of life	NQF 0210; CMS Consensus Core Set	397	10 (8-11)	49 (31-93)	0.44 (0.33-0.60)
Lung cancer EOL summary	NA	397	27 (25-30)	49 (31-93)	0.56 (0.45-0.71)
CRC (n = 87 total practices)					
Proportion of patients who died who did not enroll in hospice >3 d before death	NQF 0215; NQF 0216	87	41 (39-43)	28 (23-39)	0.26 (0.23-0.33)
>1 ED visit in last 30 d of life	NQF 0213	87	19 (17-20)	28 (23-39)	0.17 (0.15-0.23)
ICU visit in last 30 d of life	NQF 0213	87	24 (21-26)	28 (23-39)	0.37 (0.33-0.45)
Chemotherapy in last 2 wk of life	NQF 0210	87	7^d^	28 (23-39)	Could not be calculated^e^
CRC EOL summary	NA	87	23 (21-24)	28 (23-39)	0.30 (0.26-0.37)
Breast cancer (n = 23 total practices)					
Proportion of patients who died who did not enroll in hospice >3 d before death	NQF 0215; NQF 0216	23	45^d^	25 (22-36)	Could not be calculated^e^
>1 ED visit in last 30 d of life	NQF 0213	23	20 (20-20)	25 (22-36)	0.02 (0.01-0.02)
ICU visit in last 30 d of life	NQF 0213	23	27 (24-29)	25 (22-36)	0.31 (0.28-0.39)
Chemotherapy in last 2 wk of life	NQF 0210	23	10 (10-10)	25 (22-36)	0.01 (0.01-0.02)^f^
Breast cancer EOL summary	NA	23	26 (25-26)	25 (22-36)	0.11 (0.10-0.15)
For all cancers (n = 450 total practices)					
Proportion of patients who died who did not enroll in hospice >3 d before death	NQF 0215; NQF 0216	450	46 (42-51)	55 (33-104)	0.59 (0.46-0.73)
>1 ED visit in last 30 d of life	NQF 0213	450	22 (20-23)	55 (33-104)	0.30 (0.21-0.45)
ICU visit in last 30 d of life	NQF 0213	450	30 (26-34)	55 (33-104)	0.62 (0.50-0.76)
Chemotherapy in last 2 wk of life	NQF 0210	450	10 (8-11)	55 (33-104)	0.46 (0.34-0.62)
All cancers EOL summary	NA	450	27 (24-29)	55 (33-104)	0.61 (0.48-0.75)
**Survival**					
Lung cancer (n = 502 total practices)					
1 y	NA	502	51 (50-53)	60 (34-116)	0.27 (0.17-0.42)
CRC (n = 347 total practices)					
1 y	NA	347	83 (83-84)	46 (31-80)	0.18 (0.13-0.27)
CRC and lung cancer (n = 596 total practices)					
1 y	NA	596	62 (61-63)	75 (40-157)	0.29 (0.18-0.46)

Abbreviations: CMS, Centers for Medicare & Medicaid Services; CoC MASTRT, Commission on Cancer Mastectomy With Radiation Therapy; CRC, colorectal cancer; ED, emergency department; EOL, end of life; ER, estrogen receptor; ICU, intensive care unit; NA, not applicable; NCCN, National Comprehensive Cancer Network; NQF, National Quality Forum; NSCLC, non–small cell lung cancer; OCM, Oncology Care Model; PR, progesterone receptor; QOPI, Quality Oncology Practice Initiative.

^a^ Rates were calculated with multilevel hierarchical linear models adjusted for patient age, sex, race/ethnicity, marital status, year of diagnosis, Charlson Comorbidity Index, cancer stage (if more than 1 stage), census tract median household income, and census tract proportion of residents without a high school education unless otherwise noted.

^b^ Mean was used instead of median because of low number of practices with more than 20 patients.

^c^ Measure was omitted from additional analyses and summary measure because of the extremely high performance across all practices.

^d^ Mean is presented because practice-level estimates could not be modeled due to limited variation in dependent variable across practices.

^e^ Model would not run; the estimated G matrix was not positive-definite.

^f^ Model failed to converge with all variables; therefore it was run without race/ethnicity and marital status.

We studied the quality of care delivered by medical oncology practices. Patients were attributed to practices on the basis of outpatient evaluation and management visit claims with a cancer diagnosis and a CMS specialty code of medical oncology, hematology/oncology, hematology, or gynecologic oncology (eAppendix 1 and eTable 2 in the Supplement). We attributed patients to the practice (identified using tax identification numbers) with the most evaluation and management visits or with the greatest payments in the event of a tie. Patients were attributed to practices according to care in the 6 months after diagnosis for process and survival measures. Attribution for ED visits and hospitalizations focused on 6-month episodes triggered by chemotherapy, as was done for the OCM attribution.^4^ Attribution for EOL measures focused on care in the last 6 months of life. For each measure and cancer type, we identified the number of practices with at least 20 patients over the 5-year study period. For each measure, we excluded practices with fewer than 20 patients during the 5-year study period (eAppendix 2 and eTable 3 in the Supplement have additional details).

### Statistical Analysis

For each measure for which a patient was eligible, we assessed whether the measure was met. We also created summary process measures by summing the number of measures met divided by the number of measures for which patients were eligible. Utilization summary measures assessed if patients had an ED visit or hospitalization. Adjusted practice-level quality measures were estimated using multilevel hierarchical linear models with practice-level random effects to compare rates for each measure, holding patient case mix constant (eAppendix 4 in the Supplement). Models adjusted for age (65-74 years, 75-84 years, or ≥85 years), sex, race/ethnicity (White; Black; Hispanic; or other, including Asian/Pacific Islander, American Indian/Alaskan Native, and unknown), marital status (unmarried, married, or unknown), census-tract median household income (quartiles), census-level proportion of residents without a high school education (quartiles), Charlson Comorbidity Index^12^ (0, 1, 2, or ≥3), and year of diagnosis (eTable 4 in the Supplement). We adjusted for cancer stage (I, II, III, IV, or unknown) as defined by the American Joint Commission on Cancer *Cancer Staging Manual*, 7th edition, when patients with more than 1 stage classification were included in a measure. Data on race/ethnicity were missing for less than 1% of patients; these patients were classified as part of the other or unknown race/ethnicity category. Marital status was missing for approximately 5% of patients, and cancer stage was missing for 2.6% to 3.8% (depending on cancer type) of patients, who were categorized separately.

Using estimated random effects from the hierarchical linear model, we estimated the adjusted practice-level rates of each measure for each practice with at least 20 eligible patients for that measure across all study years. We assessed the reliability of measures^13,14,15^ to understand how well each measure distinguished the differences in quality across practices attributable to true variation rather than chance. Reliability represents a measure’s reproducibility and is a function of the measure’s within and between-practice variation and the sample size. Reliability was calculated as the between-practice variance divided by (between-practice variance + within-practice variance ÷ n). For each individual and summary measure, we identified reliability for the practice with the median and 25th and 75th percentile number of patients. We considered reliability lower than 0.75 (which meant that >25% of variation in a practice’s performance was associated with chance instead of true differences in quality) to be inadequate.^16,17^

Reliability calculations were based on sample sizes for newly diagnosed fee-for-service Medicare beneficiaries aged 65 years or older who were treated in the oncology practices over a 5-year period. We recalculated reliability after estimating the total number of newly diagnosed patients with cancer that a practice would be expected to treat, assuming that data on quality could be extracted from a comprehensive electronic medical record. We estimated the number of patients per practice expected if we also had data for patients with Medicare Advantage (based on SEER-Medicare data) and for individuals younger than 65 years (based on the age distribution for each cancer type [eAppendix 5 and eTable 6 in the Supplement]). In addition, we estimated reliability on the basis of the expected number of patients if 2 years of data instead of 5 years were pooled given that programs assessing quality would be interested in more recent data. These calculations assumed that between-practice performance variation would not change. However, it is possible that such variation would be larger in a more diverse population, and thus we may be underestimating reliability.

Next, using Spearman correlation coefficients, we assessed the practice-level correlations of different types of summary measures (process, utilization, EOL care, and survival) within cancer types for practices with 20 or more eligible patients for each pair of summary measures. Similarly, we assessed the correlations of summary measure types (process, utilization, EOL care, and survival) across cancer types for practices with 20 or more patients for each pair of cancer type.

The analyses were descriptive. Spearman correlation coefficients were reported with 2-sided *P* values, and *P* < .05 was considered statistically significant; however, even with statistically significant correlations, the focus was on the correlation coefficient, which was considered negligible if lower than 0.20, weak if 0.20 to 0.39, moderate if 0.40 to 0.69, and strong if 0.70 or higher. Data analyses were conducted from January 2018 to December 2020. We used SAS, version 9.2 (SAS Institute Inc), and R, version 3.5.2 (R Foundation for Statistical Computing).

## Results

We assessed care processes for 49 715 patients with lung cancer who were treated in 502 practices with 20 or more patients with lung cancer, 21 692 patients with CRC who were treated in 347 practices with 20 or more patients with CRC, and 52 901 patients with breast cancer who were treated in 492 practices with 20 or more patients with breast cancer (eTable 3 in the Supplement). Patients were 65 years or older, and approximately 50% of the patients with lung cancer and CRC and all of the patients with breast cancer were women (eTable 3 in the Supplement). The number of practices that were eligible for each process measure varied substantially. For lung cancer, among the 502 practices with 20 or more patients with cancer of any stage, only 12 to 111 practices had 20 or more patients who were eligible for each process measure (Table 1). Similarly, small numbers of practices had 20 or more patients who were eligible for various CRC and breast cancer measures (47 to 223 of 347 practices for CRC measures, and 2 to 492 of 492 practices for breast cancer measures) (Table 1).

Reliability of measures across practices was low (Table 1). Among process measures, 0 of 6 measures for lung cancer, 0 of 6 for CRC, and 3 of 11 for breast cancer (including summary measures) had reliability of 0.75 or higher for the median-sized practice (Table 1), although 2 lung cancer measures had reliability higher than 0.70. Low reliability resulted from both lack of variation in rates of the measures across practices and relatively few patients who were eligible for the measure. For example, the median (interquartile range [IQR]) rate of adjuvant chemotherapy within 60 days of surgery for patients with stage II or IIIA lung cancer was 88% (86%-90%) for the median-sized practice, and the median (IQR) number of patients was only 24 (20-34), even among the 20 practices with 20 or more patients who were eligible for this measure. Both factors contributed to reliability: 0.34 for the median-sized practice, and 0.42 for the practice at the 75th percentile in size (34 eligible patients). Reliability was higher for measures that assessed imaging (eg, reliability of 0.74 [0.69-0.80] for surveillance computed tomography for resected stage I, II, or IIIA non–small cell lung cancer, or reliability of 0.74 [0.66-0.80] for resected stage II-III CRC), which had more variation across practices and larger sample sizes. Reliability also was low for utilization, EOL care, and survival measures, in which sample sizes were generally larger but variability across practices was limited (Table 1).

**Figure 1.**
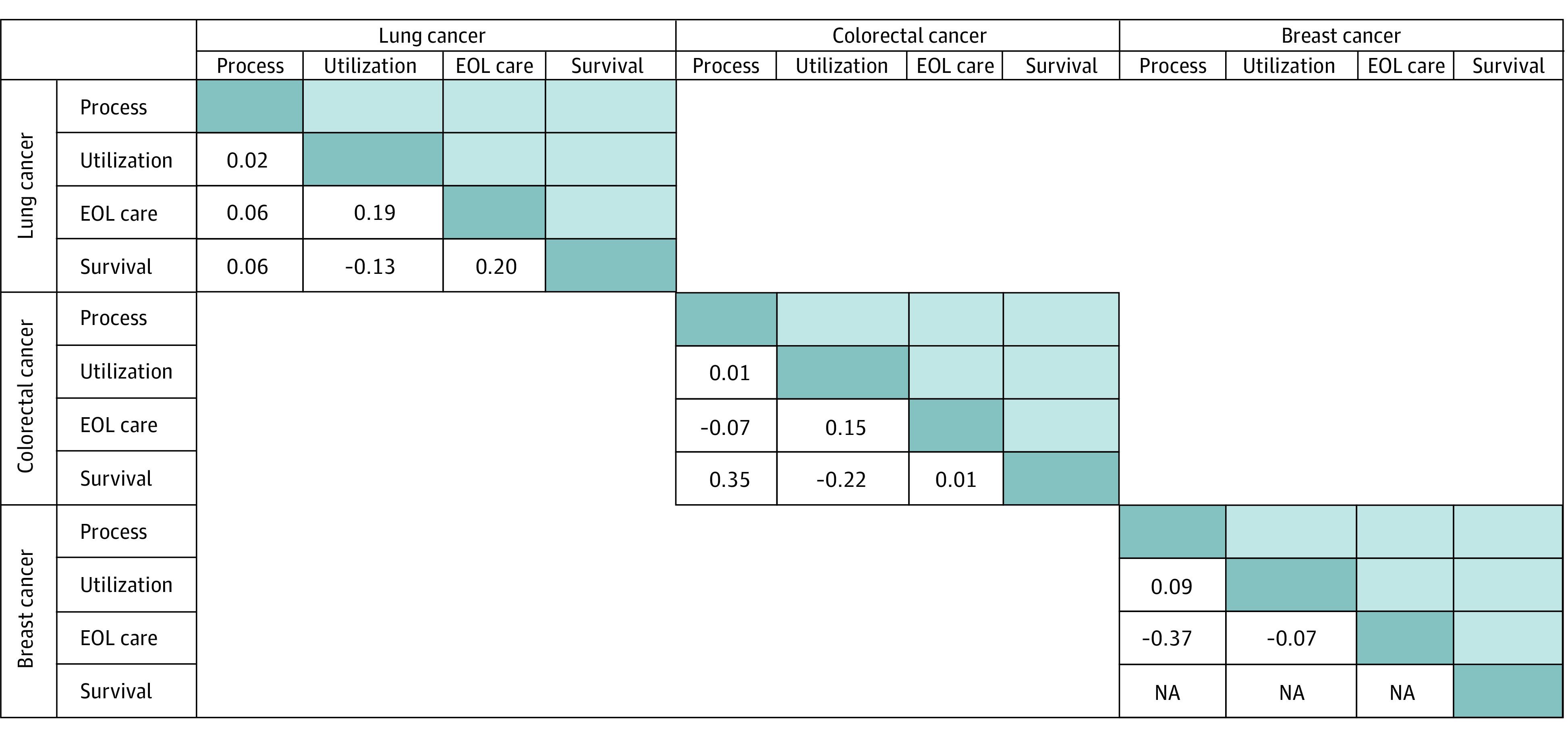
Spearman Correlation Coefficients Across Summary Measures at the Practice Level for Each Cancer Type Correlation coefficients for lung cancer were observed among 191 practices with summary process and utilization measures, 191 practices with summary process and end-of-life (EOL) care measures, 191 practices with summary process and survival measures, 375 practices with summary utilization and EOL care measures, 410 practices with summary utilization and survival measures, and 397 practices with summary EOL care and survival measures. Correlations for colorectal cancer were observed among 208 practices with summary process and utilization measures, 87 practices with summary process and EOL care measures, 227 practices with summary process and survival measures, 87 practices with summary utilization and EOL care measures, 251 practices with summary utilization and survival measures, and 87 practices with summary EOL care and survival measures. Correlations for breast cancer were observed among 434 practices with summary process and utilization measures, 23 practices with summary process and EOL care measures, and 23 practices with summary utilization and EOL care measures. NA indicates not applicable.

**Figure 2.**
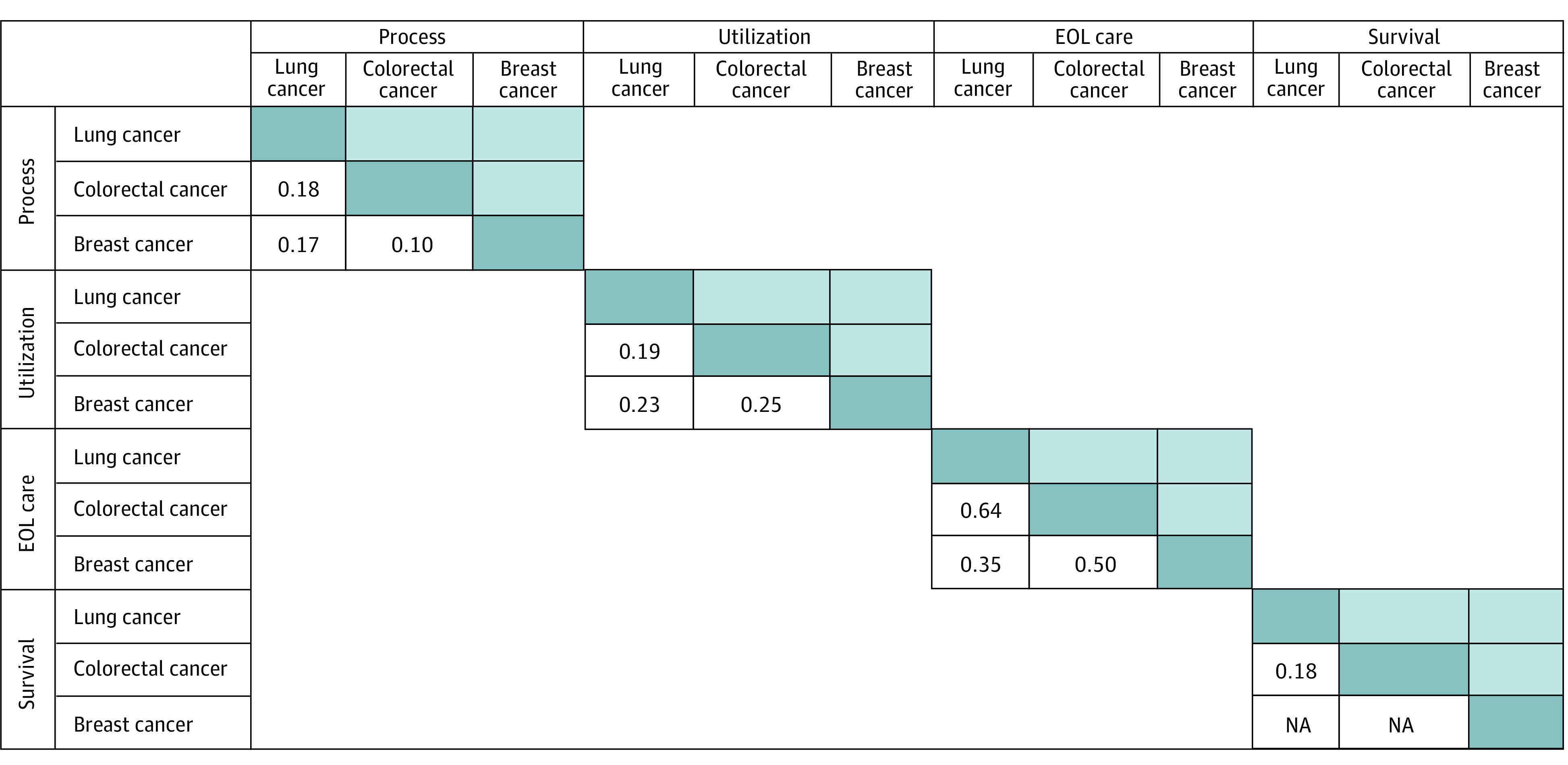
Spearman Correlation Coefficients Across Cancer Types at the Practice Level for Each Summary Measure Correlation coefficients among process measures were observed among 171 practices with at least 20 patients with lung cancer and colorectal cancer (CRC), 191 practices with at least 20 patients with lung cancer and breast cancer, and 226 practices with at least 20 patients with breast cancer and CRC. Correlations among utilization measures were observed among 259 practices with at least 20 patients with lung cancer and CRC, 382 practices with at least 20 patients with lung cancer and breast cancer, and 259 practices with at least 20 patients with breast cancer and CRC. Correlations among end-of-life care measures were observed among 87 practices with at least 20 patients with lung cancer and CRC, 23 practices with at least 20 patients with lung cancer and breast cancer, and 23 practices with at least 20 patients with breast cancer and CRC. Correlations among survival measures were observed among 342 practices with at least 20 patients with lung cancer and CRC. NA indicates not applicable.

When assessing the correlations of summary measures (process, utilization, EOL care, survival) for each cancer type, we found that the correlation across measures was low (*r* ≤ 0.21 for all), except for the correlation of the process and survival summary measures for CRC (*r* = 0.35; *P* < .001) (Figure 1 and eFigure 1 in the Supplement) and that some measures were inversely correlated, although the inverse correlation between process and EOL care measures for breast cancer was based on only 23 practices. We also observed limited correlation of process, utilization, or survival measures across cancer types. For example, summary process measures had limited or no correlation across lung cancer, breast cancer, and CRC (*r* ≤ 0.16 for all). The EOL care measures were moderately correlated across cancer types (*r* = 0.35 to 0.64) (Figure 2 and eFigure 2 in the Supplement).

Reliability estimates were based on newly diagnosed fee-for-service Medicare beneficiaries aged 65 years or older. Because reliability is a function of sample size, we recalculated reliability after estimating the sample sizes for practices, assuming that patients with Medicare Advantage and those younger than 65 years were included, as if we had data from a comprehensive electronic medical record. Table 2 shows the number of measures with an estimated reliability of 0.75 or higher with updated sample sizes to approximate all patients with newly diagnosed cancers in the practices over 5 years. Despite the much larger sample sizes, only 4 of 6 lung cancer, 1 of 6 CRC, and 7 of 11 breast cancer process measures were estimated to have reliability of 0.75 or higher, pooling 5 years of data (Table 2 and eTable 7 in the Supplement). If limited to 2 years of patients with newly diagnosed cancer instead of 5 years, then 0 of 6 lung cancer, 1 of 6 CRC, and 4 of 11 breast cancer process measures would be expected to have reliability of 0.75 or higher. None of the utilization, EOL care, or survival measures would have reliability of 0.75 or higher. eTable 2 in the Supplement shows the minimum number of patients across practices that is necessary to have reliability of 0.75 or higher across all measures.

**Table 2.** Number of Process Measures With an Estimated Reliability of 0.75 or Greater

Cohort	Original cohort of FFS Medicare beneficiaries aged ≥65 y treated by practices over 5-y study period			Assuming all patients of all ages and insurance types were treated					
				By practices over 5-y study period^a^			By practices over 2-y study period^b^		
	Range across measures		No. of measures with ≥0.75 reliability	Range across measures		No. of measures with ≥0.75 reliability	Range across measures		No. of measures with ≥0.75 reliability
	Sample size	Reliability		Sample size	Reliability		Sample size	Reliability	
**Lung cancer**									
Process	24-34	0.34-0.74	0 of 6	56-81	0.55-0.87	4 of 6	22-32	0.33-0.73	0 of 6
Utilization	80-80	0.37-0.48	0 of 3	190-190	0.58-0.69	0 of 3	76-76	0.36-0.47	0 of 2
EOL care	49-49	0.27-0.57	0 of 5	116-116	0.46-0.76	2 of 5	46-46	0.26-0.56	0 of 5
Survival	60	0.27	0 of 1	142	0.47	0 of 1	57	0.26	0 of 1
**Colorectal cancer**									
Process	28-36	0.02-0.74	0 of 6	77-99	0.06-0.89	1 of 6	31-40	0.03-0.76	1 of 6
Utilization	70-70	0.29-0.36	0 of 3	193-193	0.53-0.61	0 of 3	77-77	0.31-0.39	0 of 3
EOL care	28-28	0.17-0.37	0 of 4	77-77	0.37-0.62	0 of 4	31-31	0.19-0.40	0 of 4
Survival	46	0.18	0 of 1	127	0.37	0 of 1	51	0.19	0 of 1
**Breast cancer**^c^									
Process	24-64	0.04-0.87	3 of 11	91-242	0.12-0.96	7 of 11	36-97	0.05-0.91	4 of 11
Utilization	134-134	0.35-0.43	0 of 3	506-506	0.67-0.74	0 of 3	202-202	0.44-0.53	0 of 3
EOL care	25-25	0.01-0.31	0 of 4	94-94	0.04-0.63	0 of 4	38-38	0.02-0.40	0 of 4

Abbreviations: EOL, end-of-life; FFS, fee-for-service.

^a^ Based on the sample size from the original cohort extrapolated to include younger patients (based on the proportion of patients aged 65 y or older diagnosed and the proportion of Medicare beneficiaries enrolled in Medicare Advantage).

^b^ Based on the sample size from previous columns multiplied by 0.40.

^c^ Survival for breast cancer was not calculated because women rarely die of breast cancer within 1 year.

## Discussion

In this study of quality measures for oncology care in patients with newly diagnosed cancer across a variety of domains, we identified that most measures, including measures endorsed by national organizations, were not well suited for assessing care quality across medical oncology practices. These measures were limited by low reliability (ability to detect signal instead of noise) because of generally small sample sizes and/or limited variability in measure rates across practices, despite pooling the data for patients with newly diagnosed cancer over 5 years and after excluding practices with the smallest sample sizes. We also observed limited to no correlation of measures across measure and cancer types.

Cancer is prevalent in the United States, with more than 1.8 million individuals expected to be diagnosed in 2021.^18^ Interest in improving care for patients with cancer and the emergence of value-based payment models to reimburse that care have increased interest in measuring the quality of cancer care; however, numerous challenges exist in quality measurement. First, cancer consists of not just one but many diseases; even within cancer types, the extent of disease and tumor characteristics necessitate different local and/or systemic treatments.

Second, cancer care is multidisciplinary; individual patients may receive care from surgeons, medical oncologists, radiation oncologists, and/or palliative care clinicians. Even when guidelines recommend multidisciplinary care, it is often unclear who should be accountable for that care.^19^ For example, if a patient with early-stage estrogen receptor–positive breast cancer does not receive endocrine therapy after lumpectomy and radiation, it may be unclear whether the lack of treatment should be attributed to the patient’s surgeon, radiation oncologist, and/or medical oncologist.

Third, given the heterogeneity of cancer diagnoses, the number of patients with newly diagnosed cancers of particular type and stage who are treated at even a relatively large practice within a year tends to be small.^20^ Another effort to assess the quality of oncology care across practices in Washington state similarly found relatively small sample sizes (even after restricting to a few larger practices) and limited variability across measures.^21,22^ That effort successfully created summary measures that reflected the receipt of recommended treatment across cancer types.^21,22^ Despite larger sample sizes, reliability of the summary measures in the present study remained limited. Moreover, the lack of correlation of process measures across cancer type for the Medicare patients in the present study suggests that such a strategy is unlikely to be successful in this population.

Fourth, the available data used to analyze the quality of cancer care have limitations. Administrative data are easily available to payers but lack clinical details, such as stage, histologic type, and tumor markers. These details are critical for risk-adjusting the outcome measures and understanding the appropriateness of care. Such data will be even more important as cancer therapy becomes increasingly targeted. Medical records data provide details about cancer characteristics and care delivered, such as those collected for the ASCO Quality Oncology Practice Initiative.^23^ However, medical record abstraction is time consuming and costly. Ideally, electronic medical record systems would allow for timely and efficient quality measurement. Unfortunately, the ability to incorporate needed data into structured fields that can be combined across various electronic health record systems has yet to be realized, although this is an area of interest (eg, mCODE and CancerLinQ from ASCO, The MITRE Corporation, and others).^24^

Fifth, even among the few measures that have been endorsed by national organizations such as the National Quality Forum and CMS,^8,9^ the endorsement process has not involved assessing reliability at the practice level. Research assessing hospital and surgeon profiling for 3 Commission on Cancer surgery measures noted acceptable reliability for some but not all measures.^25^ Previous work has incorporated assessments of institution-level variation in identifying quality improvement targets^26,27,28^; such assessments are particularly important for value-based payment models that include financial incentives for practices to improve the care they deliver. Some measures had high rates of performance but little variability, whereas others had opportunities for improvement; however, we observed little variability across practices, reducing the reliability of these measures. A caveat to this analysis is that we focused on patients with newly diagnosed cancer. Nevertheless, other research has shown little change in practice-level quality over time for many measures, including measures with wide performance gaps.^29^

The limited correlations we observed across measure type suggest that these different measures provide different information or incomplete pictures of quality. For example, although the process measures reflect care that has been correlated with better long-term survival, such improvements may not be captured in the survival measure at 1 year. Similarly, inadequate risk adjustment could potentially explain limited or negative correlations between the utilization and survival measures (eg, sicker patients have more hospitalizations and worse survival).

Findings of this study may be disappointing for proponents of value-based payment and those seeking to pay for quality and not volume. Nevertheless, these findings provide a reminder of the need to assess measure reliability, particularly for high-stakes purposes such as public reporting or payment. It is possible that other measures, such as guideline-recommended use of supportive care drugs for patients undergoing chemotherapy (eg, appropriate use of prophylactic growth factors), may perform better than the measures that we examined given that they can be assessed across large populations of patients with different cancer types. Other measures worth exploring could target the avoidance of low-value care, including choice of lower cost but equally effective treatments, such as biosimilar products. However, a key factor in the choice of measures is the intended use. If measures are to be used for value-based payment models, in which incentives for practices to provide more efficient care already exist, then quality measures to assess the underuse of care would be of higher priority than measures of efficiency. Efficiency measures might be more appropriate for use by payers that are looking to identify preferred practices for their patients to receive treatment.

Additional research is needed to identify reliable quality measures for practice-level alternate payment models. For example, strategies that target supplemental data collection on a subset of practices that perform below certain thresholds on certain core measures hold promise.^30^ Given the challenges and limitations of measure-focused approaches to improving care, initiatives that aim to improve care by more productively leveraging professionalism^31^ are also promising.

### Strengths and Limitations

This study has several strengths. First, it is a comprehensive assessment of a variety of quality measures across 3 cancer types and 4 domains. Second, it analyzed hundreds of practices that treat patients with cancer and assessed measures that have been endorsed by national organizations.

This study also has some limitations. First, we used SEER linked to Medicare administrative data to identify stage-specific care for patients with a new cancer diagnosis, for whom the receipt of high-quality care may be particularly relevant, but our assessments may not generalize to the care of patients with recurrent disease. In addition, the small sample sizes that we identified may be less relevant to programs such as the OCM, which focuses on patients undergoing chemotherapy. Nevertheless, claims-based measures used in value-based programs, such as the OCM (ED visits and hospice enrollment for ≥3 days), had limited variability across practices, necessitating large sample sizes for adequate reliability, although variation may be greater in cohorts beyond patients who were recently diagnosed. We analyzed data from older adults enrolled in fee-for-service Medicare. Even after extrapolating the sample sizes to consider all patients (of all ages and insurance types) with a new diagnosis, the study results suggested that reliability would remain low, although variation in performance would likely be higher in a more diverse population.

Second, attributing patients to oncology practices can be challenging. Some patients receive care from more than 1 practice, and for many measures, the recommendations from surgeons, radiation oncologists, and primary care clinicians may be a factor in a patient’s receipt of certain therapies. We studied medical oncologists to compare similar types of practices and used established methods to identify the practices that are most important in a patient’s care.^4^

Third, we considered reliability of 0.75 or higher to be good. This decision was based on Consumer Assessment of Healthcare Providers and Systems reporting for Medicare, in which star ratings are not reported if reliability is very low (<0.60) and are reported as “low reliability” if reliability is 0.60 or higher but lower than 0.75.^32^ Although reliability of 0.90 or higher may be deemed high,^16^ others have proposed that reliability higher than 0.70 is acceptable for drawing conclusions about groups.^17^ Nevertheless, most of the measures we analyzed had reliability well below 0.70.

Fourth, we used a regression modeling approach to adjust the case mix, which relies on extrapolation of data. An alternative approach is template matching,^33^ which can avoid extrapolation but may exclude practices with patient populations that are not similar to other practices.

## Conclusions

In this cross-sectional study, we found that measures of oncology care process, utilization, EOL care, and survival that were assessed using registry and administrative data were limited by a small number of fee-for-service Medicare patients with newly diagnosed cancer across oncology practices, even after pooling 5 years of data. Most measures had low reliability because of small sample sizes and/or limited variability across practices. Moreover, the measures had limited to no correlation across measure and cancer types. Future research is needed to identify reliable quality measures for practice-level alternate payment models.

## Notes

Supplement. **eTable 1.** Consort Diagram for Study Population **eAppendix 1.** Attribution of Patients to Practices **eTable 2.** Breast, Colorectal, and Lung Cancer Diagnosis Codes **eAppendix 2.** Patient and Practices Sample Sizes and Patient Characteristics **eTable 3.** Patient and Practice Ns **eTable 4.** Patient Characteristics **eAppendix 3.** Quality Measures Specification **eTable 5.** Specification of Measures **eAppendix 4.** Modeling of Measures **eFigure 1.** Practice-Level Correlations Across Summary Measures for Each Cancer Type **eFigure 2.** Practice-Level Correlations Across Cancer Types for Each Summary Measure **eAppendix 5.** Estimation of Sample Sizes for All Newly Diagnosed Cancer Patients in Practices **eTable 6.** Estimation of Total Number of Newly Diagnosed Cancer Patients in a Practice **eTable 7.** Minimum Sample Size of Practices for Reliability ≥0.75 Based on Observed Variation and Estimated N (%) Practices with Sufficient Sample Size for Reliability ≥0.75 With Extrapolation to All Patients in Practice
