# Supplementary material for: Evaluation of Reliability and Correlations of Quality Measures in Cancer Care
Source: JAMA Netw Open. 2021 Mar 22;4(3):e212474. doi: 10.1001/jamanetworkopen.2021.2474 (PMC7985722; doi:10.1001/jamanetworkopen.2021.2474)
Supplement: Supplement. — eTable 1. Consort Diagram for Study Population eAppendix 1. Attribution of Patients to Practices eTable 2. Breast, Colorectal, and Lung Cancer Diagnosis Codes eAppendix 2. Patient and Practices Sample Sizes and Patient Characteristics eTable 3. Patient and Practice Ns eTable 4. Patient Characteristics eAppendix 3. Quality Measures Specification eTable 5. Specification of Measures eAppendix 4. Modeling of Measures eFigure 1. Practice-Level Correlations Across Summary Measures for Each Cancer Type eFigure 2. Practice-Level Correlations Across Cancer Types for Each Summary Measure eAppendix 5. Estimation of Sample Sizes for All Newly Diagnosed Cancer Patients in Practices eTable 6. Estimation of Total Number of Newly Diagnosed Cancer Patients in a Practice eTable 7. Minimum Sample Size of Practices for Reliability ≥0.75 Based on Observed Variation and Estimated N (%) Practices with Sufficient Sample Size for Reliability ≥0.75 With Extrapolation to All Patients in Practice [file jamanetwopen-e212474-s001.pdf]

Supplementary Online Content

Keating NL, Cleveland JLF, Wright AA, et al. Evaluation of reliability and correlations of quality measures in cancer care. *JAMA Netw Open*. 2021;4(3):e212474. doi:10.1001/jamanetworkopen.2021.2474

eTable 1. Consort Diagram for Study Population

eAppendix 1. Attribution of Patients to Practices

eTable 2. Breast, Colorectal, and Lung Cancer Diagnosis Codes

eAppendix 2. Patient and Practices Sample Sizes and Patient Characteristics

eTable 3. Patient and Practice Ns

eTable 4. Patient Characteristics

eAppendix 3. Quality Measures Specification

eTable 5. Specification of Measures

eAppendix 4. Modeling of Measures

eFigure 1. Practice-Level Correlations Across Summary Measures for Each Cancer Type

eFigure 2. Practice-Level Correlations Across Cancer Types for Each Summary Measure

eAppendix 5. Estimation of Sample Sizes for All Newly Diagnosed Cancer Patients in Practices

eTable 6. Estimation of Total Number of Newly Diagnosed Cancer Patients in a Practice

eTable 7. Minimum Sample Size of Practices for Reliability ≥0.75 Based on Observed Variation and Estimated N (%) Practices with Sufficient Sample Size for Reliability ≥ 0.75 With Extrapolation to All Patients in Practice

This supplementary material has been provided by the authors to give readers additional information about their work.

© 2021 Keating NL et al. *JAMA Network Open.*

eTable 1. Consort Diagram for Study Population

Inclusion Criteria	Lung	Colorectal	Breast
Patients diagnosed with cancer in 2011-2015	194,750	125,626	197,504
Patients with non-missing month of diagnosis	194,029	125,041	196,767
Patients not identified by autopsy or death certificate	189,016	123,586	195,674
Patients with cancer stage information	181,220	117,708	193,162
Patients who are not stage 0*	180,769	110,061	---
Patients continuously enrolled in Parts A and B of FFS Medicare for 6 months after diagnosis or until death	105,071	55,773	87,237
Patients without end-stage renal disease	104,601	55,445	86,841
Patients who are aged 65+	95,635	51,385	79,617
Patients who are female (breast)	---	---	78,736
**Process Measures Analyses**			
Patients with visits to a medical oncology TIN and eligible for process or survival measures	53,603	25,840	56,884
Patients eligible for process measures treated at a practice with 20+ patients eligible for process measures	49,715	23,345	52,90
**Utilization Measures Analyses**			
Patients with visits to a medical oncology TIN and eligible for utilization measures (receipt of chemotherapy)	39,111	19,682	57,599
Patients eligible for utilization measures treated at a practice with 20+ patients eligible for utilization measures	33,277	14,125	46,703
**End-of-Life Measures Analyses**			
Patients with visits to a medical oncology TIN and eligible for end-of-life measures (died during study period)	34,275	6,900	3,662
Patients eligible for end-of-life measures treated at a practice with 20+ patients eligible for endo-of-life measures	30,259	3,158	776
**Survival Measures Analyses**			
Patients with visits to a medical oncology TIN and eligible for survival measures	53,603	25,840	
Patients eligible for process measures treated at a practice with	49,715	23,345	

© 2021 Keating NL et al. *JAMA Network Open.*

20+ patients eligible for process measures			

*The breast cancer cohort initially focused on patients with invasive breast cancer only.

© 2021 Keating NL et al. *JAMA Network Open.*

eAppendix 1. Attribution of Patients to Practices

We attributed patients to medical oncology practices (based on Tax Identification Numbers) in the 6 months after diagnosis based on evaluation and management (E&M) claims for outpatient physician visits with a cancer diagnosis. Outpatient visits were identified based on Current Procedural Terminology (CPT) codes 99201-99215, 99241-99245. We required the claim to include the specific cancer diagnosis codes as described in eTable 2.

© 2021 Keating NL et al. *JAMA Network Open.*

eTable 2. Breast, Colorectal, and Lung Cancer Diagnosis Codes

Cancer	Diagnosis Codes
Breast	Icd-9: 174xx,175xx
	Icd-10: C50011,C50012,C50019,C50111,C50112,C50119,C50211,C50212,C50219,
	C50311,C50312,C50319,C50411,C50412,C50419,C50511,C50512,C50519,
	C50611,C50612,C50619,C50811,C50812,C50819,C50911,C50912,C50919
	C50021,C50022,C50029,C50121,C50122,C50129,C50221,C50222,C50229,
	C50321,C50322,C50329,C50421,C50422,C50429,C50521,C50522,C50529,
	C50621,C50622,C50629,C50821,C50822,C50829,C50921,C50922,C50929
Colorectal	Icd-9: 153xx 154xx
	Icd-10: C180-C20x
Lung	Icd-9: 162xx
	Icd-10: C3400 ,C3401 ,C3402 ,C3410 ,C3411 ,C3412 ,C342 ,C3430 ,C3431 ,C3432 , C3480 ,C3481 ,C3482 ,C3490 ,C3491 ,C3492

Patients were assigned to the medical oncology practice with the most E&M cancer-related visits; if there was a tie, we assigned patients to the practice with the most recent visit. Identification of visits to a "medical oncology practice" was based on E&M visit code with a CMS specialty code of medical oncology (83), hematology/oncology (90), hematology (82), or gynecologic oncology (98). These are the specialties that typically provide chemotherapy to patients. Although gynecologic oncologists are surgeons by training, they often prescribe chemotherapy to women with gynecologic (and sometimes breast) cancers.

© 2021 Keating NL et al. *JAMA Network Open.*

eAppendix 2. Patient and Practices Sample Sizes and Patient Characteristics

Because our analysis was focused on comparing measures across oncology practices, we required at least 20 patients over the 5-year period so that we would have a minimal number of patients from which to estimate practice-level rates of the measure. We chose to use 20 patients because the Centers for Medicare & Medicaid Services uses a similar threshold of 20 patients in a year for public reporting for its Hospital Compare program. (see Hospital Inpatient Specifications Manuals. Version 5.10 – Discharges 07/01/2021 through 12/31/2021. Accessed on January 10, 2020 at https://qualitynet.cms.gov/inpatient/specifications-manuals).

eTable 3 demonstrates the total number of patients attributed to a medical oncology practice and the total number of practices as well as the N's after limiting to practices with at least 20 patients over the study period. As can be seen, and depending on the measure type, while fewer than half of practices had at least 20 patients, the vast majority of patients (e.g., >90% for process measures) were treated at a practice with at least 20 patients of the relevant type. Smaller proportions were seen for the end of life measures for lung and colorectal cancer, but this is because few patients with these cancers die within 5 years of diagnosis.

© 2021 Keating NL et al. *JAMA Network Open.*

eTable 3. Patient and Practice Ns

Cohort	N practices total	N patients total	N practices with 20+ patients	N patients in practices with 20+ patients	Mean (SD) N patients in practices (20+ pts)	Median (25th, 75th percentiles) N patients in practices (20+ pts)	Minimum, Maximum N patients in practices (20+ pts)
**Process Measures**							
Lung cancer process	1,315	53,603	502	49,715	99 (128)	60 (34,116)	20-1877
Colorectal cancer process	1,110	25,840	347	23,345	67 (72)	46 (31-80)	20-961
Breast cancer process	1,333	56,884	492	52,901	108 (138)	64 (36,124)	20-1921
**Utilization Measures**							
Lung cancer utilization	2,464	39,111	421	33,277	79 (99)	51 (30,90)	20-1430
Colorectal cancer utilization	2,015	19,682	262	14,125	54 (55)	38 (27,63)	20-693
Breast cancer utilization	4,809	57,599	498	46,703	94 (125)	55 (31,113)	20-1798
All cancers utilization	6,194	114,758	701	101,432	145 (229)	72 (36,173)	20-3872
**End-of-Life Measures**							
Lung cancer end-of-life	1,217	34,275	397	30,259	76 (91)	49 (31,93)	20-1268
Colorectal cancer end-of-life	848	6,900	87	3,158	36 (30)	28 (23,39)	20-263
Breast cancer end-of-life	668	3,262	23	776	34 (25)	25 (22,36)	20-137
All cancers end-of-life	1,324	44,388	450	40,203	89 (113)	55 (33,104)	20-1666
**Survival Measures**							
Lung cancer survival	1,315	53,603	502	49,715	99 (128)	60 (34,116)	20-1877
Colorectal cancer survival	1,110	25,840	347	23,345	67 (72)	46 (31-80)	20-961
Lung & colorectal cancer survival	1,434	81,185	596	77,285	130 (181)	75 (40,157)	20-2833

© 2021 Keating NL et al. *JAMA Network Open.*

eTable 4. Patient Characteristics

	Process/Survival Cohorts			Utilization Cohorts			End of Life Cohorts		
**Characteristic**	**Lung**	**CRC**	**Breast**	**Lung**	**CRC**	**Breast**	**Lung**	**CRC**	**Breast**
Age at diagnosis, N (%)									
65-74 years	25,633 (51.6%)	10,905 (46.7%)	30,028 (56.8%)	19,173 (57.6%)	7,595 (53.8%)	28,667 (61.4%)	15,333 (50.7%)	1,364 (43.2%)	353 (45.5%)
75-84 years	19,478 (39.2%)	9215 (39.5%)	17,419 (32.9%)	12,228 (36.8%)	5,284 (37.4%)	14,438 (30.9%)	11,993 (39.6%)	1,290 (40.9%)	285 (36.7%)
85+ years	4,604 (9.3%)	3225 (13.8%)	5,454 (10.3%)	1,876 (5.6%)	1,246 (8.8%)	3,598 (7.7%)	2,933 (9.7%)	504 (16.0%)	138 (17.8%)
Sex, N (%)									
Male	25,173 (50.6%)	11,403 (48.9%)		17,072 (51.3%)	7,255 (51.4%)		16,222 (53.6%)	1,583 (50.1%)	
Female	24,542 (49.4%)	11,942 (51.2%)	52,901 (100%)	16,205 (48.7%)	6,870 (48.6%)	46,703 (100%)	14,037 (46.4%)	1,575 (49.9%)	776 (100%)
Race/Ethnicity, N (%)									
White	41,657 (83.8%)	18,770 (80.4%)	43,727 (82.7%)	28,086 (84.4%)	11,288 (79.9%)	38,480 (82.4%)	25,454 (84.1%)	2,449 (77.6%)	630 (81.2%)
Black	3,710 (7.5%)	1,838 (7.9%)	3,782 (7.2%)	2,320 (7.0%)	1,183 (8.4%)	3,402 (7.3%)	2,306 (7.6%)	327 (10.4%)	104 (13.4%)
Hispanic	1,865 (3.7%)	1,385 (6.0%)	2,699 (5.1%)	1,195 (3.6%)	844 (6.0%)	2,546 (5.5%)	1,156 (3.8%)	203 (6.4%)	27 (3.5%)
Asian/Pacific Islander	2,279 (4.6%)	1,197 (5.1%)	2,269 (4.3%)	1,555 (4.7%)	738 (5.2%)	1,991 (4.3%)	1,240 (4.1%)	163 (5.2%)	14 (1.8%)
Other	204 (0.4%)	155 (0.7%)	424 (0.8%)	121 (0.4%)	72 (0.5%)	284 (0.6%)	103 (0.3%)	16 (0.5%)	1 (0.1%)
Marital Status, N (%)									
Married	26,464 (53.2%)	12,329 (52.8%)	25,305 (47.8%)	19,008 (57.1%)	7,944 (56.2%)	22,685 (48.6%)	16,084 (53.2%)	1,567 (49.6%)	273 (35.2%)
Single/divorced/separated/widowed	21,329 (42.9%)	9,977 (42.7%)	24,992 (47.2%)	13,202 (39.7%)	5,619 (39.8%)	22,131 (47.4%)	13,046 (43.1%)	1,473 (46.6%)	469 (60.4%)
Unknown	1,922 (3.9%)	1,039 (4.5%)	2,604 (4.9%)	1,067 (3.2%)	562 (4.0%)	1,887 (4.0%)	1,129 (3.7%)	118 (3.7%)	34 (4.4%)
Census-tract median household income (quartiles)									
Quartile 1	13,063 (26.3%)	5,727 (24.5%)	10,912 (20.6%)	8,446 (25.4%)	3,460 (24.5%)	10,160 (21.8%)	8,150 (26.9%)	684 (21.7%)	183 (23.6%)

© 2021 Keating NL et al. *JAMA Network Open.*

	Process/Survival Cohorts			Utilization Cohorts			End of Life Cohorts		
**Characteristic**	**Lung**	**CRC**	**Breast**	**Lung**	**CRC**	**Breast**	**Lung**	**CRC**	**Breast**
Quartile 2	12,912 (26.0%)	5,994 (25.7%)	12,648 (23.9%)	8,517 (25.6%)	3,522 (24.9%)	10,906 (23.4%)	8,015 (26.5%)	810 (25.7%)	192 (24.7%)
Quartile 3	12,618 (25.4%)	5,929 (25.4%)	13,938 (26.4%)	8,405 (25.3%)	3,538 (25.1%)	11,906 (25.5%)	7,525 (24.9%)	838 (26.5%)	199 (25.6%)
Quartile 4	11,095 (22.3%)	5,683 (24.3%)	15,361 (29.0%)	7,893 (23.7%)	3,599 (25.5%)	13,701 (29.3%)	6,554 (21.7%)	825 (26.1%)	202 (26.0%)
Census tract % high school graduates									
Quartile 1	11,221 (22.6%)	5,602 (24.0%)	15,601 (29.5%)	7,882 (23.7%)	3,458 (24.5%)	13,723 (29.4%)	6,690 (22.1%)	808 (25.6%)	214 (27.6%)
Quartile 2	12,577 (25.3%)	5,987 (25.7%)	13,897 (26.3%)	8,406 (25.3%)	3,555 (25.2%)	11,962 (25.6%)	7.529 (24.9%)	817 (25.9%)	198 (25.5%)
Quartile 3	12,928 (26.0%)	5,918 (25.4%)	12,718 (24.0%)	8,463 (25.4%)	3,536 (25.0%)	11,056 (23.7%)	8,008 (26.5%)	775 (24.5%)	208 (26.8%)
Quartile 4	12,967 (26.1%)	5,827 (25.0%)	10,649 (20.1%)	8,513 (25.6%)	3,570 (25.3%)	9,939 (21.3%)	8,020 (26.5%)	757 (24.0%)	156 (20.1%)
Charlson, N (%)									
0	9,373 (18.9%)	7,674 (32.9%)	28,802 (54.5%)	6,995 (21.0%)	5,002 (35.4%)	25,553 (54.7%)	5,621 (18.6%)	947 (30.0%)	322 (41.5%)
1	14,216 (28.6%)	6,031 (25.8%)	12,710 (24.0%)	10,078 (30.3%)	3,825 (27.1%)	11,435 (24.5%)	8,336 (27.6%)	803 (25.4%)	186 (24.0%)
2	10,092 (20.3%)	3,745 (16.0%)	5,597 (10.6%)	6,700 (20.1%)	2,221 (15.7%)	4,944 (10.6%)	6,089 (20.1%)	516 (16.3%)	96 (12.4%)
3+	16,034 (32.3%)	5,895 (25.3%)	5,792 (11.0%)	9,504 (28.6%)	3,077 (21.8%)	4,771 (10.2%)	10,213 (33.8%)	892 (28.3%)	172 (22.2%)
Year of Diagnosis, N (%)									
2011	9,699 (19.5%)	4,464 (19.1%)	9,928 (18.8%)	6786 (20.4%),	2,855 (20.2%)	9,257 (19.8%)	6,471 (21.4%)	710 (22.5%)	180 (23.2%)
2012	10,283 (20.7%)	4,736 (20.3%)	10,504 (19.9%)	7,064 (21.2%)	2,877 (20.4%)	9,610 (20.6%)	6,948 (23.0%)	740 (23.4%)	203 (26.2%)
2013	9,980 (20.1%)	4,602 (19.7%)	10,731 (20.3%)	6,652 (20.0%)	2,831 (20.0%)	9,502 (20.4%)	6,446 (21.3%)	677 (21.4%)	182 (23.5%)
2014	9,922 (20.0%)	4,815 (20.6%)	10,797 (20.4%)	6,536 (19.6%)	2,806 (19.9%)	9,319 (20.0%)	5,771 (19.1%)	611 (19.4%)	125 (16.1%)
2015	9,831 (19.8%)	4,728 (20.3%)	10,941 (20.7%)	6,239 (18.8%)	2,756 (19.5%)	9,015 (19.3%)	4,623 (15.3%)	420 (13.3%)	86 (11.1%)
Stage									

© 2021 Keating NL et al. *JAMA Network Open.*

	Process/Survival Cohorts			Utilization Cohorts			End of Life Cohorts		
**Characteristic**	**Lung**	**CRC**	**Breast**	**Lung**	**CRC**	**Breast**	**Lung**	**CRC**	**Breast**
1	9,743 (19.6%)	3,493 (15.0%)	25,681 (48.6%)	4,930 (14.8%)	1,264 (9.0%)	20,713 (44.4%)	2,022 (6.7%)	79 (2.5%)	41 (5.3%)
2	2,858 (5.8%)	6,690 (28.7%)	15,781 (29.8%)	2,224 (6.7%)	2,812 (19.9%)	14,275 (30.6%)	899 (3.0%)	275 (8.7%)	122 (15.7%)
3	13,238 (26.6%)	7,748 (33.2%)	4,447 (8.4%)	10,095 (30.3%)	5,873 (41.6%)	4,506 (9.7%)	7,847 (25.9%)	616 (19.5%)	138 (17.8%)
4	22695 (45.7%)	5,083 (21.8%)	2,687 (5.1%)	15,206 (45.7%)	3,931 (27.8%)	2,490 (5.3%)	18,913 (62.5%)	2,162 (68.5%)	455 (58.6%)
Unknown	1,122 (2.3%)	296 (1.3%)	1,077 (2.0%)	763 (2.3%)	216 (1.5%)	749 (1.6%)	558 (1.8%)	24 (0.8%)	17 (2.2%)
SEER Registry, N (%)									
San Francisco/San Jose	2577 (5.2%)	1,271 (5.4%)	3,144 (5.9%)	1,749 (5.3%)	817 (5.8%)	2,916 (6.2%)	1,567 (5.2%)	190 (6.0%)	1 (0.1%)
Connecticut	3,107 (6.3%)	1,365 (5.9%)	3,161 (6.0%)	2,110 (6.3%)	778 (5.5%)	2,996 (6.4%)	1,926 (6.4%)	178 (5.6%)	45 (5.8%)
Detroit	3,489 (7.0%)	1,372 (5.9%)	3,086 (5.8%)	2,182 (6.6%)	828 (5.9%)	2,566 (5.5%)	2,069 (6.8%)	199 (6.3%)	95 (12.2%)
Hawaii	447 (0.9%)	251 (1.1%)	589 (1.1%)	207 (0.6%)	106 (0.8%)	440 (0.9%)	276 (0.9%)	28 (0.9%)	
Iowa	3,164 (6.4%)	1,755 (7.5%)	2,978 (5.6%)	2,083 (6.3%)	899 (6.4%)	2,748 (5.9%)	1,943 (6.4%)	214 (6.8%)	45 (5.8%)
New Mexico	788 (1.6%)	342 (1.5%)	1,081 (2.0%)	493 (1.5%)	213 (1.5%)	762 (1.6%)	521 (1.7%)	43 (1.4%)	
Seattle	3,067 (6.2%)	1,389 (6.0%)	3,339 (6.3%)	1,832 (5.5%)	724 (5.1%)	2,129 (4.6%)	1,953 (6.5%)	214 (6.8%)	30 (3.9%)
Utah	569 (1.1%)	367 (1.6%)	1,201 (2.3%)	342 (1.0%)	237 (1.7%)	913 (2.0%)	354 (1.2%)	39 (1.2%)	22 (2.8%)
Los Angeles	2,460 (5.0%)	1,398 (6.0%)	3,260 (6.2%)	1,773 (5.3%)	950 (6.7%)	3,001 (6.4%)	1,359 (4.5%)	214 (6.8%)	36 (4.6%)
Greater California	8,435 (17.0%)	4,135 (17.7%)	10,381 (19.6%)	5,566 (16.7%)	2,366 (16.8%)	8,945 (19.2%)	4,992 (16.5%)	531 (16.8%)	74 (9.5%)
Kentucky	4,672 (9.4%)	1,689 (7.2%)	3,299 (6.2%)	3,292 (9.9%)	1,080 (7.7%)	3,246 (7.0%)	2,993 (9.9%)	116 (3.7%)	46 (5.9%)
Louisiana	3,115 (6.3%)	1,512 (6.5%)	2,744 (5.2%)	2,154 (6.5%)	1,082 (7.7%)	2,770 (5.9%)	1,894 (6.3%)	197 (6.2%)	47 (6.1%)
New Jersey	7,269 (14.6%)	3,667 (15.7%)	8,237 (15.6%)	5,066 (15.2%)	2,244 (15.9%)	7,678 (16.4%)	4,238 (14.0%)	523 (16.6%)	180 (23.2%)

© 2021 Keating NL et al. *JAMA Network Open.*

	Process/Survival Cohorts			Utilization Cohorts			End of Life Cohorts		
**Characteristic**	**Lung**	**CRC**	**Breast**	**Lung**	**CRC**	**Breast**	**Lung**	**CRC**	**Breast**
Georgia	6,556 (13.2%)	2,832 (12.1%)	6,401 (12.1%)	4,428 (13.3%)	1,801 (12.8%)	5,593 (12.0%)	4,174 (13.8%)	472 (15.0%)	155 (20.0%)

Note that models did not include registry because they included random effects for practice.

© 2021 Keating NL et al. *JAMA Network Open.*

eAppendix 3. Quality Measures Specification

eTable 5 includes details on the specification of the various measures. Note that the process measures are based on care that has been shown in prior studies (typically randomized clinical trials) to be associated with improved outcomes (including survival, disease-free survival, and/or progression-free survival).

© 2021 Keating NL et al. *JAMA Network Open.*

eTable 5. Specification of Measures

Measure	Cohort	Time period for treatment*
**Process measures**		
**Lung Cancer**		
Receipt of at least one treatment with radiation therapy for patients with stage I/II NSCLC who did not undergo resection	Patients with stage I/II NSLCL who did not undergo surgical resection within 180 days of diagnosis (as above). Patients were required to be alive and not in a Medicare HMO through 180 days from diagnosis.	Month of diagnosis through 6 months after diagnosis
Adjuvant chemotherapy received by patients with stage II or IIIA NSCLC who underwent resection	Patients with stage II or IIIA NSCLC who had surgical resection (as above) and were in parts A&B Medicare and survived month of diagnosis through 4 months after surgery	Month of diagnosis through 4 months after surgery
Adjuvant chemotherapy and/or radiation therapy for patients with Stage IIIA NSCLC who underwent resection	All patients with stage IIIA NSCLC who underwent surgical resection. Patients were required to be alive and not in a Medicare HMO month of diagnosis through 4 months after surgery	Month of diagnosis through 4 months after surgery
Chemo within 60 days of surgery (among patients who had surgery) §	All patients with stage IIIA NSCLC who underwent surgical resection. Patients were required to be alive and not in a Medicare HMO month of diagnosis through 6 months after surgery	Month of diagnosis through 6 months after surgery
Surveillance chest CT for patients with Stage I, II, IIIA NSCLC who underwent resection	All patients with stage I, II, IIIA NSCLC who underwent surgical resection. Patients were required to be alive and not in a Medicare HMO through 14 months from surgical resection	Date of surgery through 14 months after surgery
Lung Cancer Process Summary	Each patient in each practice coded 0/1 for each measure for which they are eligible, proportion of all measures met/all eligible	
**Colorectal Cancer**		
Adjuvant chemotherapy received within 4 months of surgery by patients with AJCC stage III colon cancer	Patients with stage III colon cancer who received colon resection (in part A & B of FFS Medicare & survived through surgery and 120 days after surgery)	Month of diagnosis through 4 months after colon resection
Adjuvant chemo within 60 days of surgery (among patients who had surgery) §	Patients with stage III colon cancer who received colon resection and at least 1 dose chemo	Month of diagnosis through 6 months after colon resection
Surveillance colonoscopy 1 year after colon resection	Patients with stage I-III colon cancer who received colon resection (in part A & B of FFS Medicare & survived through 14 mo after surgery)	Months 10-14 following surgery
Surveillance CEA testing after curative resection	Patients with stage II-III colon cancer who received colon resection (in part A & B of FFS Medicare & survived through 12 mo after surgery)	Date of surgery through 12 months

© 2021 Keating NL et al. *JAMA Network Open.*

Measure	Cohort	Time period for treatment*
At least one CT of chest/abdomen/pelvis within 12 months of resection	Patients with stage II-III colon cancer who received colon resection (in part A & B of FFS Medicare & survived through 12 mo after surgery)	Date of surgery through 12 months
Colorectal Cancer Process Summary	Each patient in each practice coded 0/1 for each measure for which they are eligible, proportion of all measures met/all eligible	
**Breast Cancer (female & male)**		
Radiation after breast conserving surgery (note: breast conserving surgery = lumpectomy)	Patients with stage I-III breast cancer who underwent breast conserving surgery (in parts A&B of FFS Medicare & survived through 12 mo after surgery)	Month of diagnosis through 12 months after surgery
Radiation after mastectomy with ≥4 positive nodes	Patients with stage II or III breast cancer who have ≥4 positive lymph nodes (in part A & B of FFS Medicare & survived through 12 mo after surgery)	Month of diagnosis through 12 months after surgery
Chemotherapy within 4 months of surgery for women under 70 with AJCC stage I (T1c or N1) to III ER/PR negative breast cancer	Patients with stage I(T1c or N1), II, or III breast cancers who have ER negative AND PR negative tumors (T1c=tumor size >10 mm; N1=node positive) (in part A & B of FFS Medicare & survived through 4 months after surgery)	Month of diagnosis through 4 months after surgery
Chemo within 60 days of surgery (among patients who had surgery) §	All patients with stage I (T1c or N1) to III ER/PR negative breast cancer who underwent surgical resection. Patients were required to be alive and not in a Medicare HMO month of diagnosis through 6 months after surgery	Month of diagnosis through 6 months after surgery
Adjuvant endocrine therapy for women with ER/PR positive cancer within 1 year	Patients with stage I(T1c or N1)-III breast cancer who underwent breast surgery who have ER positive OR PR positive breast cancers and are enrolled in Medicare Parts A, B& D & survived through 12 mo after surgery) (T1c=tumor size >10 mm, N1=node positive)	Month of diagnosis through 12 months after surgery
HER2 status assessed	All breast cancer patients, assess for HER2 (See Derived HER2 Recode variable in SEER data)	n/a
Trastuzumab received when HER2 is positive	Among breast cancer patients with HER2 positive tumors, receipt of at least 1 dose of trastuzumab within 1 year after surgery (in parts A&B of FFS Medicare & survived through 12 mo after surgery)	Month of diagnosis through 12 months after surgery
Trastuzumab not given when HER2 is negative or unknown	Among breast cancer patients with HER2 negative or unknown tumors, no receipt of trastuzumab within 1 year after surgery (in parts A&B of FFS Medicare & survived through 12 mo after surgery)	Month of diagnosis through 12 months after surgery
IV bisphosphonates or denosumab for breast cancer bone metastases	Among breast cancer patients with E&M visit (inpatient, outpatient, ED) with diagnosis code for bone metastases (see codes) within 1 year of diagnosis and in part A & B of FFS Medicare & survived through 3 months after first bone met dx	Month of 1st claim for bone met through 3 months after
PET, CT, or bone scan within 3 months of diagnosis for stage I or II breast cancer	Among breast cancer patients with stage I or II breast cancer, in part A & B of FFS Medicare & survived through 3 months after diagnosis)	

© 2021 Keating NL et al. *JAMA Network Open.*

Measure	Cohort	Time period for treatment*
Surveillance mammogram 1 year after breast cancer surgery (low rate better)	Patients with stage I-III breast cancer who had breast surgery (in part A & B of FFS Medicare & survived through 12 mo after surgery)	Months 10-14 following surgery
Breast Cancer Process Summary	Each patient in each practice coded 0/1 for each measure for which they are eligible, proportion of all measures met/all eligible	
**Utilization Measures (all cancers)**		
Number of hospital admissions in 6 month chemotherapy “episode”	Number of hospital admissions during 6-month chemotherapy “episode”	Date of first chemotherapy through 180 days
Proportion of patients with hospital admission in 6 month chemotherapy “episode”	Proportion of patients with at least one hospital admission during 6-month chemotherapy “episode”	Date of first chemotherapy through 180 days
Number of ED visits in 6 month chemotherapy “episode”	Number of ED visits or observation stays that did not result in a hospital admission during 6-month chemotherapy “episode”	Date of first chemotherapy through 180 days
Proportion of patients with ED visits in 6 month chemotherapy “episode”	Proportion of patients with ED visits or observation stays that did not result in a hospital admission during 6-month chemotherapy “episode”	Date of first chemotherapy through 180 days
**Utilization Summary Measure**	Proportion of patients with ED visit OR inpatient hospitalization	Date of first chemotherapy through 180 days
**End of Life Measures (all cancers)**		
>1 ED visit in last 30 days of life	More than one ED visit in last 30 days of life (in part A&B of FFS Medicare in last 30 days of life)	30 days before death
ICU visit in last 30 days of life	Any ICU visit in last 30 days of life (in part A&B of FFS Medicare in last 30 days of life)	30 days before death
Chemotherapy in last 2 weeks of life	Chemotherapy in last 14 days of life (in part A&B of FFS Medicare in last 30 days of life)	14 days before death
No hospice use or hospice within 3 days of death	Proportion of patients who died who did not enroll in hospice more than 3 days before death	30 days before death
End of Life Summary Measure	Each patient in each practice coded 0/1 for each measure for which they are eligible, proportion of all measures met/all eligible	
**Survival**		
Lung cancer 12-month survival	Proportion of patients surviving 12 months from date of diagnosis	
Colorectal cancer 12-month survival	Proportion of patients surviving 12 months from date of diagnosis	
Survival Summary Measure	Proportion of patients with lung or colorectal cancer surviving 12 months from date of diagnosis	

© 2021 Keating NL et al. *JAMA Network Open.*

eAppendix 4. Modeling of Measures

We used multi-level hierarchical linear models with practice-level random effects to calculate adjusted practice-level rates for each quality measure or summary measure. Models took the form:

$Y_{ij} = \beta_{0j} + \beta_{1j}X_{1j} + \epsilon_{ij}$

where Y_{ij} is receipt of the quality measure of interest for the patient i in practice j
β_{0j} is the intercept for the practice j; we assume $\beta_{0j} \sim N(0, \sigma^2_{between})$
X_{ij} a vector of covariates for patient i in practice j
ϵ_{ij} is the random error associated with the patient i in practice j; we assume $\epsilon_{ij} \sim N(0, \sigma^2_{within})$

Models adjusted for age (65-74, 75-84, ≥85), sex, race/ethnicity (White, Black, other/unknown), marital status (unmarried, married, unknown), census-tract median household income (quartiles), census-level proportion of residents without a high school education (quartiles), Charlson Comorbidity Index[12] (0, 1, 2, ≥3), and year of diagnosis. We adjusted for American Joint Commission on Cancer, 7th edition stage (1, 2, 3, 4, unknown) when patients of more than one stage were included in a measure.

Estimated practice-level random effects, β_{0j}, represent the difference in performance for practice j compared to an average practice, holding patient characteristics constant. We use these estimated random effects to compute adjusted practice-level rates as:

$Y_{j,adjusted} = Y_{ave} * \beta_{0j}$

where Y_{ave} is the mean rate for the measure averaged across all patients in the cohort.

Finally, we compute reliability for a practice with median number of attributed patients as the ratio of the between variance to the total variance:

$R_{med} = \sigma^2_{between}/(\sigma^2_{between}+\sigma^2_{within}/n_{med})$

Here we provide SAS code for the models for an example measure:

```
PROC HPMIXED NOCLPRINT DATA=CRC1_20X;
   CLASS TIN_6MODX_CANCDX CRC_STG_AJCC6GRP;
   MODEL ADJCHEMO120S = AGE7584 AGE85P FEMALE BLACK RACE_OTHER MARRIED
            MARITAL_UNKN
            CTMEDHHINCQ2-CTMEDHHINCQ4 CTMEDHHINC_UNKN CTNONHSGRADQ2-
            CTNONHSGRADQ4 CTNONHSGRAD_UNKN
            YRDX2012-YRDX2015 CHARLSON1-CHARLSON3 CRC_STG_AJCC6GRP;
   RANDOM TIN_6MODX_CANCDX / S;
```

where TIN_6MODX_CANCDX represents the attributed practice.

© 2021 Keating NL et al. *JAMA Network Open.*

eFigure 1. Practice-Level Correlations Across Summary Measures for Each Cancer Type

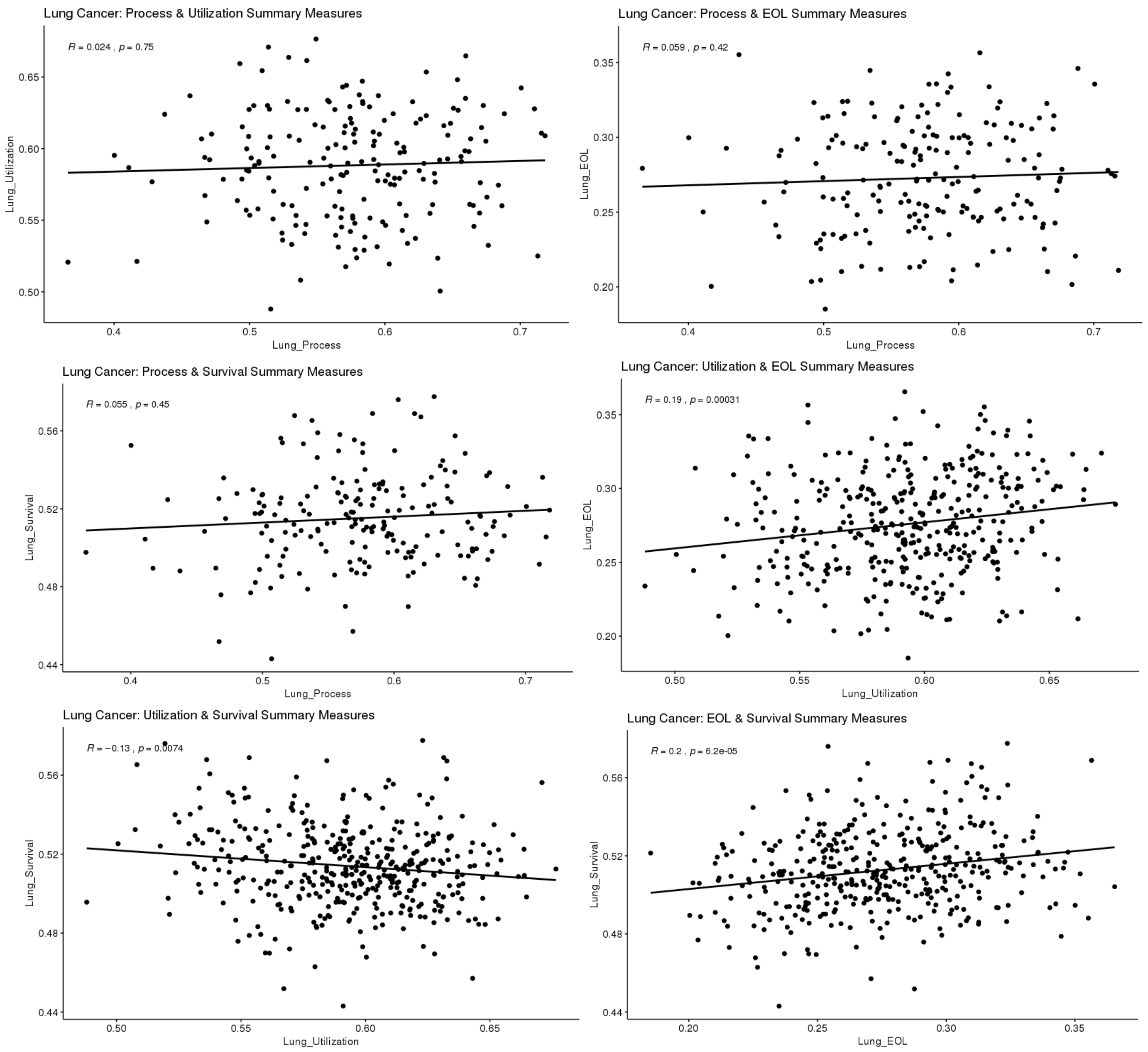

© 2021 Keating NL et al. *JAMA Network Open.*

Colorectal Cancer: Process & Utilization Summary Measures

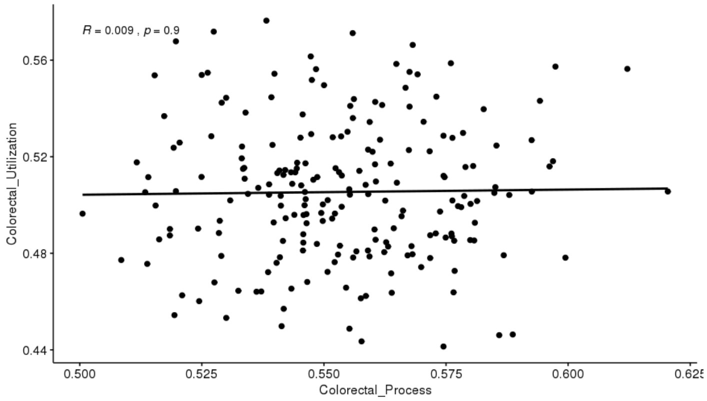

Colorectal Cancer: Process & EOL Summary Measures

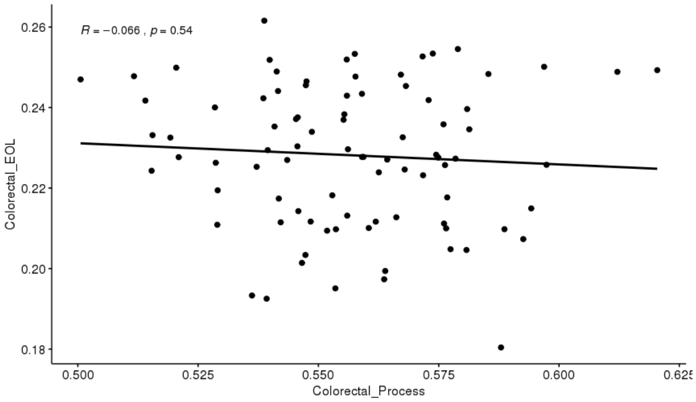

Colorectal Cancer: Process & Survival Summary Measures

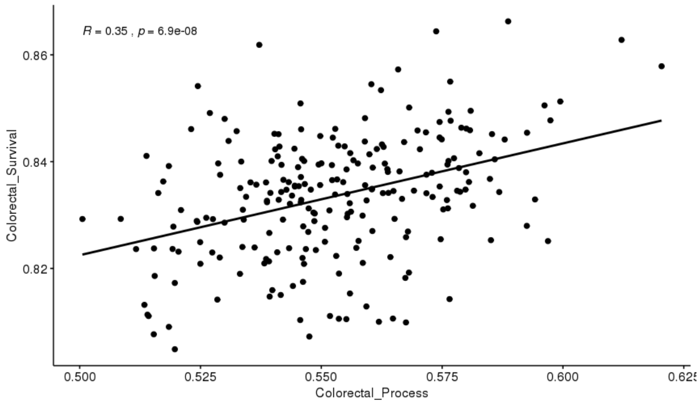

Colorectal Cancer: Utilization & EOL Summary Measures

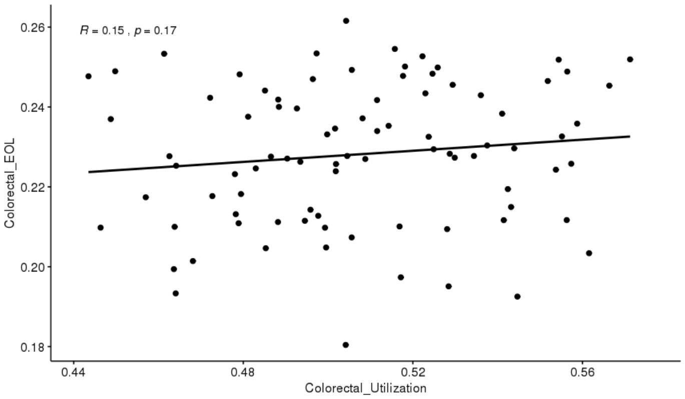

Colorectal Cancer: Utilization & Survival Summary Measures

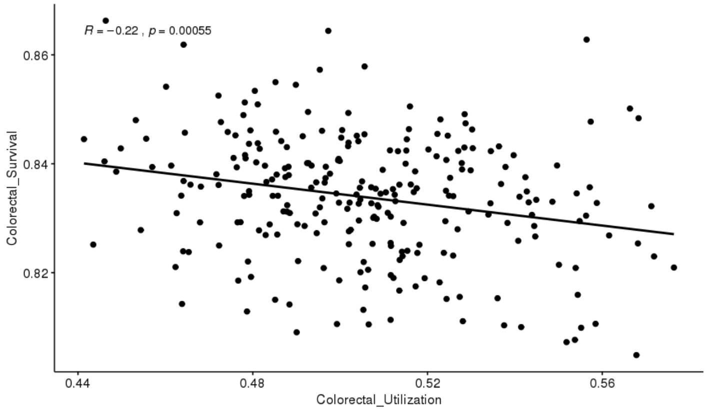

Colorectal Cancer: EOL & Survival Summary Measures

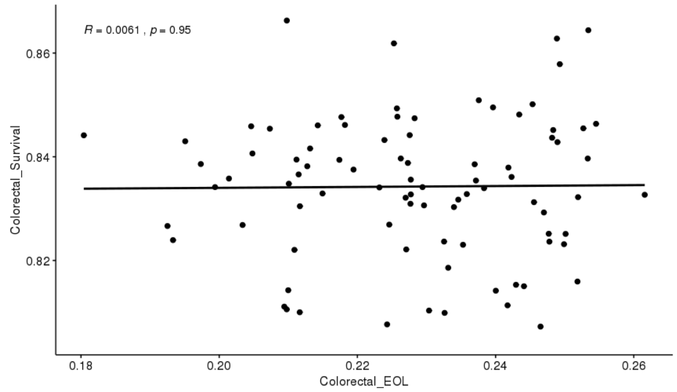

Breast Cancer: Process & Utilization Summary Measures

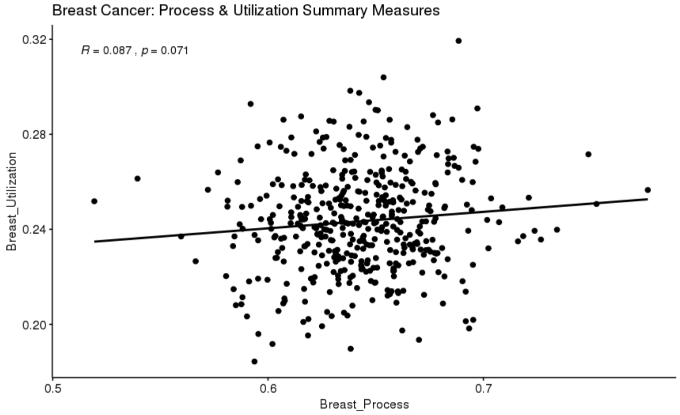

Breast Cancer: Process & EOL Summary Measures

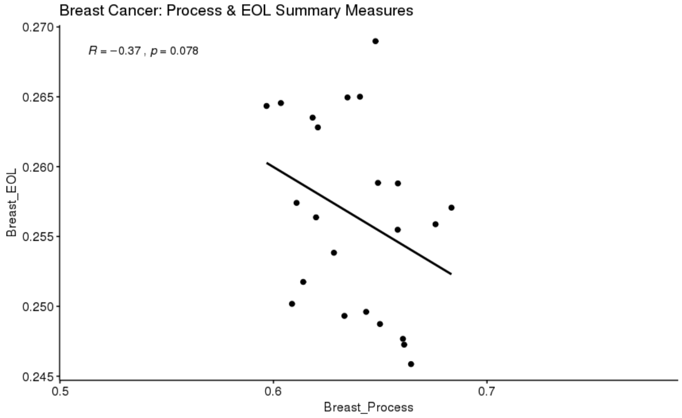

© 2021 Keating NL et al. *JAMA Network Open.*

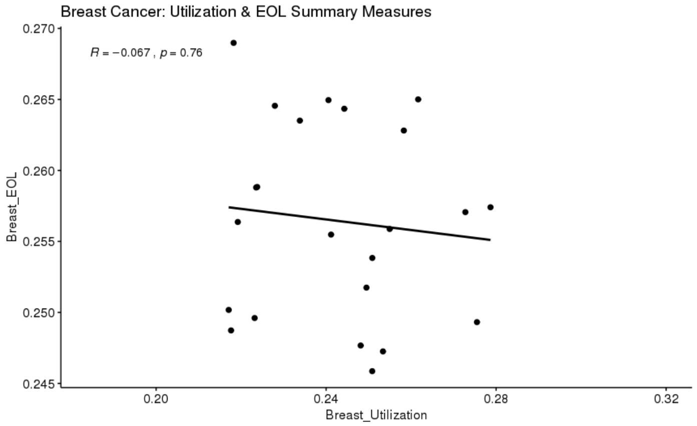

© 2021 Keating NL et al. *JAMA Network Open.*

eFigure 2. Practice-Level Correlations Across Cancer Types for Each Summary Measure

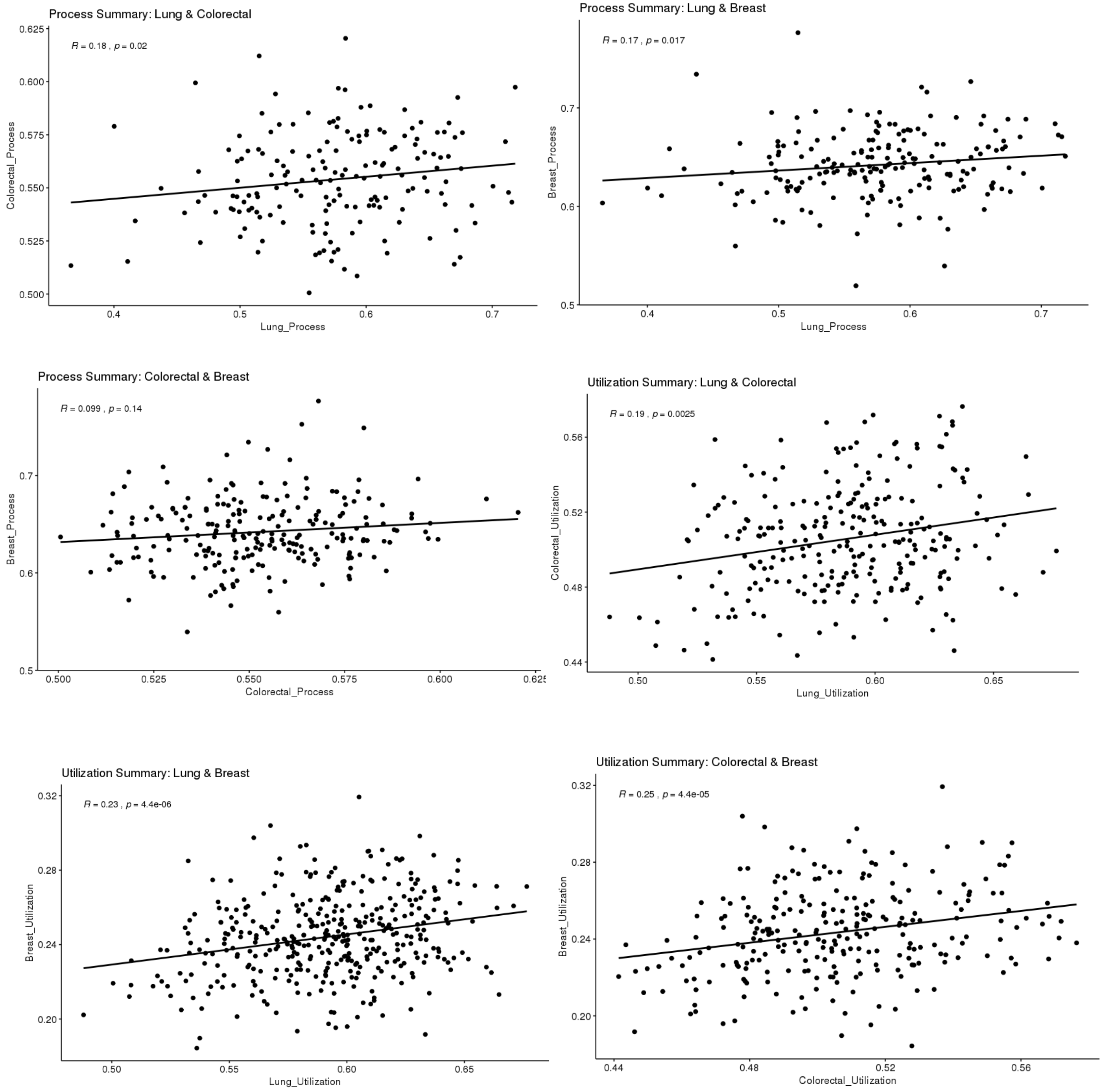

© 2021 Keating NL et al. *JAMA Network Open.*

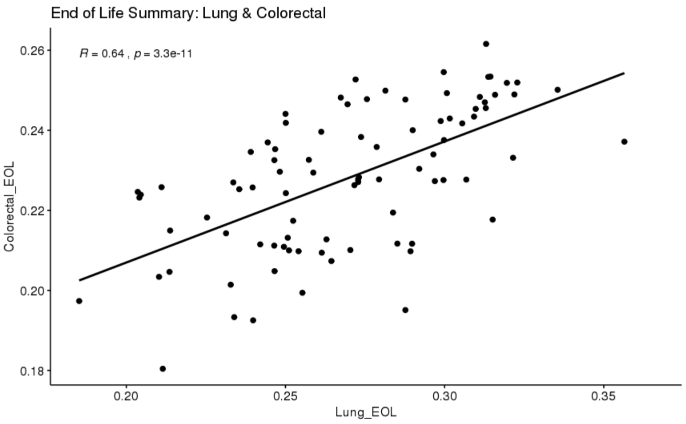

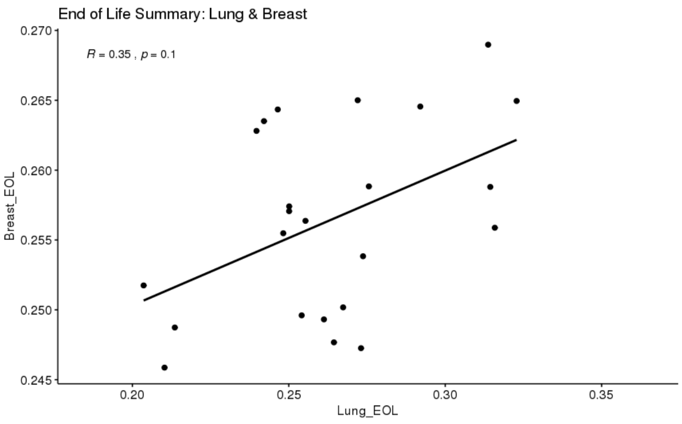

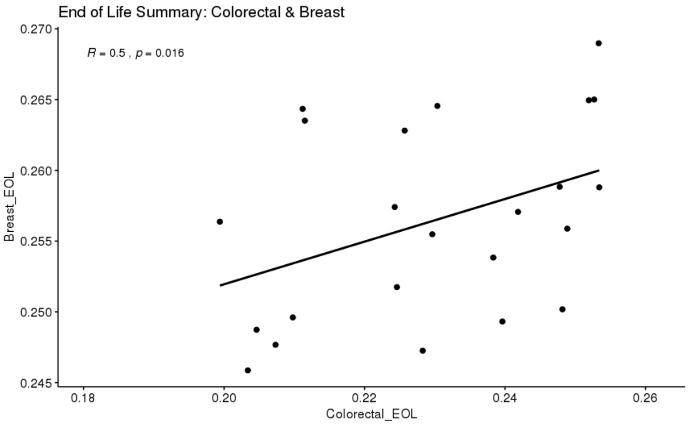

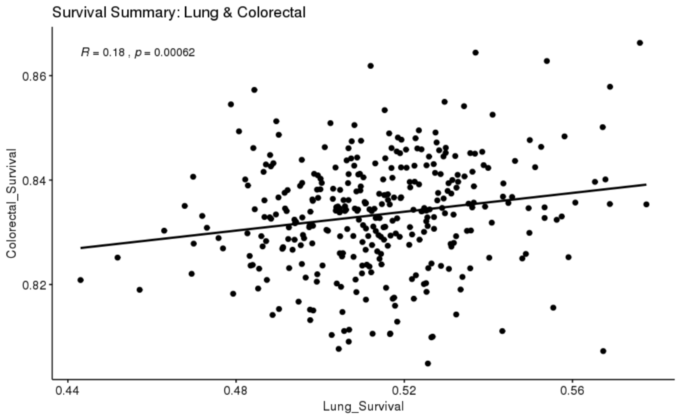

© 2021 Keating NL et al. *JAMA Network Open.*

eAppendix 5. Estimation of Sample Sizes for All Newly Diagnosed Cancer Patients in Practices

As described in the methods, we recalculated reliability after estimating the total number of newly diagnosed patients with lung, colon/rectal, or breast cancer that a practice would be expected to treat, assuming data on quality could be extracted from a comprehensive electronic medical record.

We first estimated the number of patients per practice expected if we also had data for Medicare Advantage patients (based on SEER-Medicare data) and individuals aged <65 years (based on the age distribution of each cancer type). Our first estimates assumed 5 years of data, as for the main analyses.
We first identified the total number of patients aged 65 and older in the SEER Medicare data including both fee-for-service Medicare patients and Medicare Advantage patients. This provided an estimate of the total number of patients aged 65 and older. We then used data on the median age at diagnosis for each cancer type to estimate the total number of patients aged 65+ and thus estimate the total number of patients of all ages. For example, the median age at breast cancer diagnosis is 62, thus we estimated that 45% of all breast cancer patients are aged 65+ at diagnosis. The median age at diagnosed of lung cancer is 70, so we estimated that 67% of all lung cancer patients are diagnosed at age 65+. The median age of diagnosis for colon cancer is ~70 (68 for men, 72 for women) and rectal cancer is 63 years, thus we estimated that approximately 60% of rectal cancer patients are diagnosed at age 65 and older.

We then estimated the proportion of all patients represented in our cohort (column 6 in eTable 3. For each practice, we then divided the number of patients by this factor.

© 2021 Keating NL et al. *JAMA Network Open.*

eTable 6. Estimation of Total Number of Newly Diagnosed Cancer Patients in a Practice

Cancer type	N patients all ages aged 65+ in SEER areas[a]	Estimated % of patients aged 65+[b]	Estimated N patients of all ages (column 2 x column 3)	N patients in our cohort (65+ fee-for-service Medicare)	% of all patients represented in our cohort (column 5 / column 4)
Lung cancer	152,005	67%	226,873	95,635	42.1%
Colorectal cancer	85,152	60%	141,920	51,385	36.2%
Breast cancer	133,828	45%	297,396	78,736	26.5%

[a]Based on SEER-Medicare data including Medicare Advantage patients
[b]Based on median age at diagnosis

Additionally, we estimated reliability based on the expected number of patients if we pooled 2 years of data instead of 5 years, since programs assessing quality would be most interested in more recent data.

© 2021 Keating NL et al. *JAMA Network Open.*

eTable 7. Minimum Sample Size of Practices for Reliability ≥0.75 Based on Observed Variation and Estimated N (%) Practices with Sufficient Sample Size for Reliability ≥ 0.75 with Extrapolation to All Patients in Practice

Measure	Minimum Sample Size in Practice for Reliability ≥0.75	N (%)* practices: SEER-Medicare Cohort (FFS Medicare Beneficiaries), 5 Years	N (%)* practices: Assuming All Patients Treated by a Practice, 5 Years	N (%)* practices: Assuming All Patients Treated by a Practice, 2 Years
**PROCESS MEASURES**				
**Lung Cancer – Processes (502 total practices)**				
Radiation for stage I/II NSCLC if no surgery	29	16 (3.2%)	32 (6.4%)	13 (2.6%)
Adjuvant chemo for stage II/IIIA NSCLC with surgery	53	5 (1.0%)	25 (5.0%)	4 (0.8%)
Chemo within 60 days of surgery for resected stage II/IIIA NSCLC	138	1 (0.2%)	1 (0.2%)	1 (0.2%)
Chemo and radiation for stage IIIA NSCLC	Cannot calculate			
Surveillance chest computed tomography for resected stage I/II/IIIA NSCLC	31	51 (10.2%)	111 (22.1%)	46 (9.2%)
Lung Cancer - Process Summary	76	18 (3.6%)	99 (19.7%)	18 (3.6%)
**Colorectal Cancer – Processes (347 total practices)**				
Adjuvant chemotherapy for stage III colon cancer	163	1 (0.3%)	6 (1.7%)	1 (0.3%)
Chemotherapy within 60 days of surgery for resected stage III colon cancer	186	0 (0.0%)	4 (1.2%)	1 (0.3%)
Surveillance colonoscopy for resected stage I-III colorectal cancers	4498	0 (0.0%)	0 (0.0%)	0 (0.0%)
Surveillance CEA for stage II/III colorectal cancers	149	3 (0.9%)	42 (12.1%)	5 (1.4%)
Surveillance computed tomography for resected stage II-III colorectal cancers	39	80 (23.1%)	184 (53.0%)	96 (27.7%)
Colorectal Cancer - Process Summary	214	2 (0.6%)	30 (8.6%)	3 (0.9%)
**Breast Cancer – Processes (492 total practices)**				
Radiation after breast-conserving surgery	42	184 (37.4%)	328 (66.7%)	262 (53.3%)

© 2021 Keating NL et al. *JAMA Network Open.*

Measure	Minimum Sample Size in Practice for Reliability ≥0.75	N (%)* practices: SEER-Medicare Cohort (FFS Medicare Beneficiaries), 5 Years	N (%)* practices: Assuming All Patients Treated by a Practice, 5 Years	N (%)* practices: Assuming All Patients Treated by a Practice, 2 Years
Radiation after mastectomy for high-risk cancers	Cannot calculate			
Adjuvant chemo for stage I (T1c)-III, ER/PR negative cancers	Cannot calculate			
Chemo within 60 days of surgery for stage I-III breast cancer	74	1 (0.2%)	16 (3.3%)	1 (0.2%)
Adjuvant endocrine therapy for ER/PR positive breast cancers	177	7 (1.4%)	108 (22.0%)	22 (4.5%)
HER2 assessed at diagnosis	62	260 (52.8%)	492 (100%)	338 (68.7%)
Trastuzumab received when HER2 positive	67	4 (0.8%)	66 (13.4%)	9 (1.8%)
Intravenous bisphosphonates or denosumab if bone metastases	1909	0 (0.0%)	0 (0.0%)	0 (0.0%)
PET scan, computed tomography or bone scan at diagnosis for stage I/II breast cancer (low value; lower score better)	26	374 (76.0%)	437 (88.8%)	437 (88.8%)
Surveillance mammography for stage I/II breast cancer	139	78 (15.9%)	301 (61.2%)	131 (26.6%)
Breast Cancer - Process Summary	96	171 (34.8%)	441 (89.6%)	249 (50.6%)
**UTILIZATION MEASURES****				
**Lung Cancer – Utilization (421 total practices)**				
Hospitalizations during chemotherapy episodes	259	46 (10.9%)	160 (38.0%)	39 (9.3%)
ED visit during 6-month chemotherapy episode that did not lead to hospital stay	404	18 (4.3%)	83 (19.7%)	17 (4.0%)
Lung Cancer - Utilization Summary: Hospitalization OR ED visit	295	32 (7.6%)	133 (31.6%)	27 (6.4%)
**Colorectal Cancer – Utilization (262 total practices)**				
Hospitalizations during chemotherapy episodes	518	1 (0.4%)	22 (8.4%)	1 (0.4%)
ED visit during 6-month chemotherapy episode that did not lead to hospital stay	387	7 (2.7%)	42 (16.0%)	7 (2.7%)

© 2021 Keating NL et al. *JAMA Network Open.*

Measure	Minimum Sample Size in Practice for Reliability ≥0.75	N (%)* practices: SEER-Medicare Cohort (FFS Medicare Beneficiaries), 5 Years	N (%)* practices: Assuming All Patients Treated by a Practice, 5 Years	N (%)* practices: Assuming All Patients Treated by a Practice, 2 Years
Colorectal Cancer - Utilization Summary: Hospitalization OR ED visit	369	7 (2.7%)	48 (18.3%)	7 (2.7%)
**Breast Cancer – Utilization (498 total practices)**				
Hospitalizations during chemotherapy episodes	761	25 (5.0%)	184 (36.9%)	47 (9.4%)
ED visit during 6-month chemotherapy episode that did not lead to hospital stay	592	38 (7.6%)	222 (44.6%)	78 (15.7%)
Breast Cancer - Utilization Summary: Hospitalization OR ED visit	540	42 (8.4%)	235 (47.2%)	96 (19.3%)
**All Cancers – Utilization (701 total practices)**				
Hospitalizations during chemotherapy episodes	405	155 (22.1%)	351 (50.1%)	185 (26.4%)
ED visit during 6-month chemotherapy episode that did not lead to hospital stay	447	140 (20.0%)	321 (45.8%)	165 (23.5%)
All Cancers Utilization Summary: Hospitalization OR ED visit	473	128 (18.3%)	307 (43.8%)	152 (21.7%)
**END-OF-LIFE MEASURES**				
**Lung Cancer - End-of-Life (397 total practices)**				
Proportion of patients who died who did not enroll in hospice more than 3 days before death	118	65 (16.4%)	197 (49.6%)	64 (16.1%)
>1 ER visit in last 30 days of life	405	4 (1.0%)	32 (8.1%)	4 (1.0%)
ICU visit in last 30 days of life	109	77 (19.4%)	213 (53.7%)	70 (17.6%)
Chemotherapy in last 2 weeks of life	188	30 (7.6%)	113 (28.5%)	24 (6.0%)
Lung Cancer – End-of-Life Summary	114	70 (17.6%)	199 (50.1%)	65 (16.4%)
**Colorectal Cancer (87 total practices)**				
Proportion of patients who died who did not enroll in hospice more than 3 days before death	234	1 (1.1%)	4 (4.6%)	1 (1.1%)

© 2021 Keating NL et al. *JAMA Network Open.*

Measure	Minimum Sample Size in Practice for Reliability ≥0.75	N (%)* practices: SEER-Medicare Cohort (FFS Medicare Beneficiaries), 5 Years	N (%)* practices: Assuming All Patients Treated by a Practice, 5 Years	N (%)* practices: Assuming All Patients Treated by a Practice, 2 Years
>1 ER visit in last 30 days of life	403	0 (0.0%)	1 (1.1%)	0 (0.0%)
ICU visit in last 30 days of life	141	1 (1.1%)	10 (11.5%)	1 (1.1%)
Chemotherapy in last 2 weeks of life	Cannot calculate			
Colorectal Cancer - End-of-Life Summary	200	1 (1.1%)	4 (4.6%)	1 (1.1%)
**Breast Cancer (23 total practices)**				
Proportion of patients who died who did not enroll in hospice more than 3 days before death	Cannot calculate			
>1 ER visit in last 30 days of life	4824	0 (0.0%)	0 (0.0%)	0 (0.0%)
ICU visit in last 30 days of life	169	0 (0.0%)	4 (17.4%)	1 (4.3%)
Chemotherapy in last 2 weeks of life	6587	0 (0.0%)	0 (0.0%)	0 (0.0%)
Breast Cancer - End-of-Life Summary	591	0 (0.0%)	0 (0.0%)	0 (0.0%)
**All Cancers (450 total practices)**				
Proportion of patients who died who did not enroll in hospice more than 3 days before death	117	96 (21.3%)	257 (57.1%)	96 (21.3%)
>1 ER visit in last 30 days of life	382	7 (1.6%)	63 (14.0%)	7 (1.6%)
ICU visit in last 30 days of life	100	119 (26.4%)	295 (65.6%)	119 (26.4%)
Chemotherapy in last 2 weeks of life	191	39 (8.7%)	165 (36.7%)	39 (8.7%)
All Cancers - End of Life Summary	106	110 (24.4%)	286 (63.6%)	109 (24.2%)
**Survival Measures**				
**Lung Cancer (502 total practices)**				
1-year survival	488	6 (1.2%)	53 (10.6%)	6 (1.2%)

© 2021 Keating NL et al. *JAMA Network Open.*

Measure	Minimum Sample Size in Practice for Reliability ≥0.75	N (%)* practices: SEER-Medicare Cohort (FFS Medicare Beneficiaries), 5 Years	N (%)* practices: Assuming All Patients Treated by a Practice, 5 Years	N (%)* practices: Assuming All Patients Treated by a Practice, 2 Years
**Colorectal Cancer (347 total practices)**				
1-year survival	646	1 (0.3%)	5 (1.4%)	1 (0.3%)
**Colorectal & Lung Cancer (596 total practices)**				
1-year survival	559	12 (2.0%)	89 (14.9%)	11 (1.8%)

Abbreviations: NSCLC=non-small cell lung cancer; CEA=carcinoembryonic antigen; PET=positron emission tomography
*Note: we calculated the percentages of practices out of the total number of practices eligible for each measure based on having at least 20 patients eligible in the SEER-Medicare dataset. If data on all patients were available, this number would be greater, but there also would be additional practices with small numbers of patients.
**Note: our utilization measures were based on newly diagnosed cancer patients; numbers would be much greater if looking at cancer chemotherapy episodes such as in the Oncology Care Model. In the Oncology Care Model, the mean (SD) number of attributed episodes in the 18-month baseline period was 141.3 (379) nationally, but 543 (1078) for the 190 practices participating in OCM. See First Annual Report from the Evaluation Team of the Oncology Care Model: Baseline Period: https://downloads.cms.gov/files/cmmi/ocm-baselinereport.pdf

© 2021 Keating NL et al. *JAMA Network Open.*
